# Local GABAergic signaling within sensory ganglia controls peripheral nociceptive transmission

**DOI:** 10.1172/JCI86812

**Published:** 2017-04-04

**Authors:** Xiaona Du, Han Hao, Yuehui Yang, Sha Huang, Caixue Wang, Sylvain Gigout, Rosmaliza Ramli, Xinmeng Li, Ewa Jaworska, Ian Edwards, Jim Deuchars, Yuchio Yanagawa, Jinlong Qi, Bingcai Guan, David B. Jaffe, Hailin Zhang, Nikita Gamper

**Affiliations:** 1 Department of Pharmacology, Hebei Medical University, The Key Laboratory of Neural and Vascular Biology, Ministry of Education, China; The Key Laboratory of New Drug Pharmacology and Toxicology, Hebei Province; Shijiazhuang, China.; 2 Faculty of Biological Sciences, University of Leeds, Leeds, United Kingdom.; 3 School of Dental Sciences, Universiti Sains Malaysia, Kelantan, Malaysia.; 4 Department of Genetic and Behavioral Neuroscience, Gunma University Graduate School of Medicine and Japan Science and Technology Agency, CREST, Maebashi, Japan.; 5 Department of Biology, UTSA Neurosciences Institute, University of Texas at San Antonio, San Antonio, Texas, USA.

## Abstract

The integration of somatosensory information is generally assumed to be a function of the central nervous system (CNS). Here we describe fully functional GABAergic communication within rodent peripheral sensory ganglia and show that it can modulate transmission of pain-related signals from the peripheral sensory nerves to the CNS. We found that sensory neurons express major proteins necessary for GABA synthesis and release and that sensory neurons released GABA in response to depolarization. In vivo focal infusion of GABA or GABA reuptake inhibitor to sensory ganglia dramatically reduced acute peripherally induced nociception and alleviated neuropathic and inflammatory pain. In addition, focal application of GABA receptor antagonists to sensory ganglia triggered or exacerbated peripherally induced nociception. We also demonstrated that chemogenetic or optogenetic depolarization of GABAergic dorsal root ganglion neurons in vivo reduced acute and chronic peripherally induced nociception. Mechanistically, GABA depolarized the majority of sensory neuron somata, yet produced a net inhibitory effect on the nociceptive transmission due to the filtering effect at nociceptive fiber T-junctions. Our findings indicate that peripheral somatosensory ganglia represent a hitherto underappreciated site of somatosensory signal integration and offer a potential target for therapeutic intervention.

## Introduction

Peripheral nerves convey to the central nervous system (CNS) versatile information about the body’s environment. An important function of these nerves is informing the brain about ongoing or imminent body damage, a sensation commonly known as pain. It is generally accepted that healthy peripheral nerves conduct action potentials (APs) without interruption from their respective sites of origin (i.e., at the peripheral nerve endings in the skin, viscera, muscle, etc.) to the superficial laminae of dorsal spinal cord where synaptic transmission first takes place. In the spinal cord, and subsequently in higher CNS centers, peripheral somatosensory signals are integrated and analyzed (1). It is also generally assumed that before entering the spinal cord peripheral nerve fibers receive no bona fide synaptic input and cell bodies of peripheral nerve fibers within the sensory ganglia are not necessary for AP propagation from the periphery to the spinal cord (2). Yet sensory neuron somata may contribute to pathological peripheral excitation in some chronic pain conditions (3–6).

Surprisingly, cell bodies of sensory neurons (particularly those that specifically respond to painful stimuli, the nociceptors) express multiple receptors for major neurotransmitters such as acetylcholine, glutamate, and GABA (7–10). There is currently no coherent theory for (a) why these receptors are present in sensory neuron somata; (b) what (if any) are the source(s) of neurotransmitters that activate these receptors; or (c) what physiological role activation of these receptors may play in sensory signaling. Particularly, dorsal root ganglion (DRG) neuron cell bodies express sizable GABA_A_ Cl^–^ currents (11–15). Yet the common conception is that there are no local sources of GABA to activate these receptors (16) and, thus, somatic GABA_A_ receptors are perhaps a by-product of receptor trafficking to the presynaptic terminals in the spinal cord where these mediate inhibitory “primary afferent depolarization” (17–19). Here we describe a fully functional local GABAergic transmission within the DRG. We explore its role in acute and chronic nociceptive transduction and also its potential as a therapeutic target for chronic pain treatment.

## Results

### Somatic GABA release in DRG.

**Figure 1:**
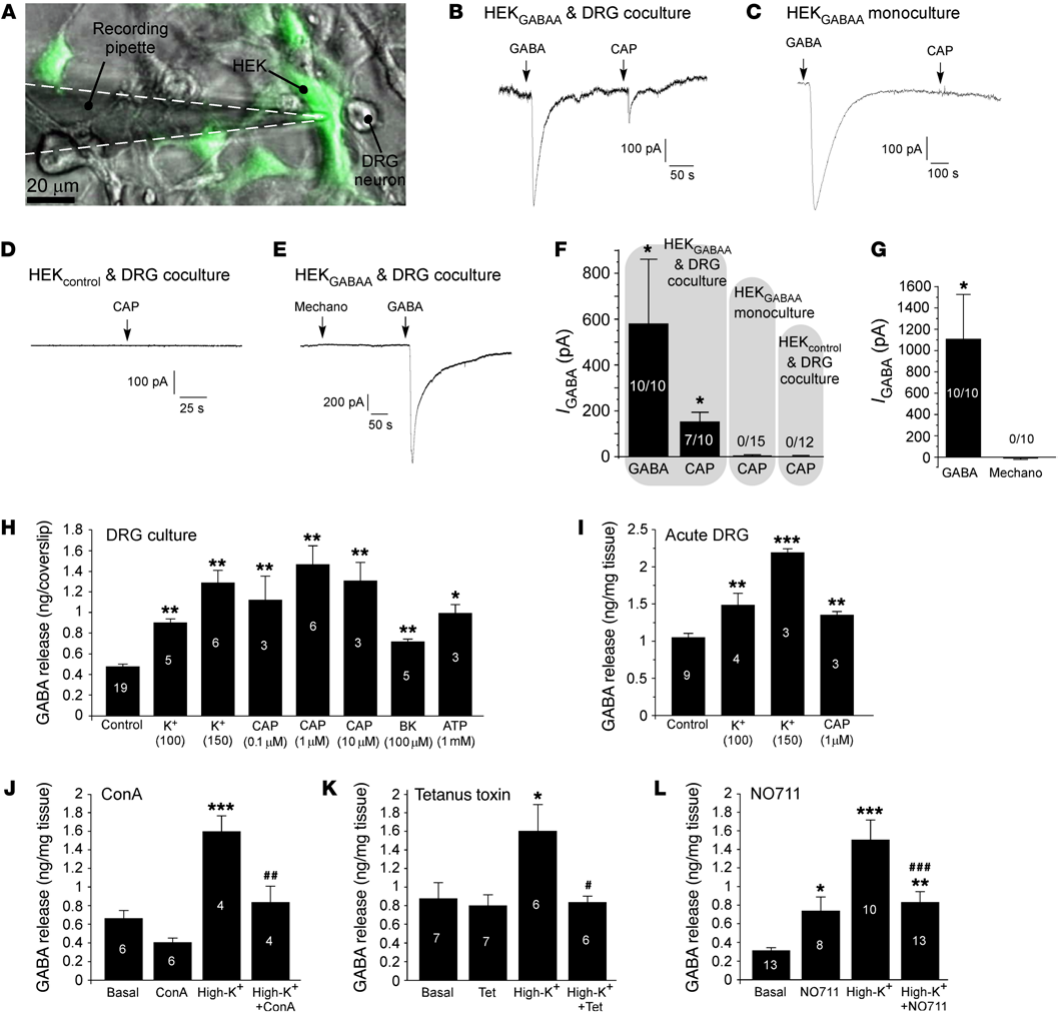
Stimulus-induced release of GABA from DRG neurons. (**A**–**E**) “Sniffing patch” experiments. (**A**) Coculture of DRG neurons with HEK293 cells transiently cotransfected with α_1_, β_2_, and γ_2_ GABA_A_ subunits and GFP (HEK_GABAA_ cells). (**B**) An example of recording from the GFP-positive HEK_GABAA_ cell juxtaposed to a small-diameter rat DRG neuron (as shown in **A**). Timing of GABA (200 μM) and capsaicin (CAP, 1 μM) applications is indicated by the arrows. (**C**) A similar experiment but with HEK_GABAA_ monoculture (no DRG neurons present). (**D**) A recording from a nontransfected HEK293 (HEK_control_) cell in close apposition to a small DRG neuron. (**E**) Mechanical stimulation of DRG neuron in HEK_GABAA_-DRG coculture did not activate inward current in HEK_GABAA_ cell. (**F**) Summary of the experiments shown in **B**–**E**; number of responsive cells from the total number of recordings is indicated within/above each bar. (**G**) Summary of experiments shown in **E**; “Mechano” indicates mechanical stimulation of DRG neuron adjacent to the “sniffing” HEK_GABAA_ cell. *Significant difference from baseline at *P* < 0.05 (1-way ANOVA with Bonferroni correction). (**H**–**L**) HPLC analyses. (**H**) Release of GABA from the dissociated DRG cultures in response to various stimuli (as indicated). (**I**) Similar to **H** but acutely extracted, non-dissociated DRGs were used. Asterisks indicate significant difference from basal release: *P < 0.05, **P < 0.01, ***P < 0.001 (1-way ANOVA with Bonferroni correction). Number of experiments is indicated within each bar. (**J**–**L**) Effects of the synaptic transmission inhibitors concanamycin A (0.5 μM; **J**) and tetanus toxin (10 μg/ml; **K**), as well as the GAT1 blocker NO711 (200 μM; **L**), on the basal and high-extracellular-K^+^-induced GABA release from acutely extracted whole DRGs. Asterisks indicate significant difference from basal release: *P < 0.05, **P < 0.01, ***P < 0.001; number symbols indicate significant difference from high-K^+^-induced release: ^#^*P* < 0.05, ^##^*P* < 0.01, ^###^*P* < 0.001 (1-way ANOVA with Bonferroni correction). Number of experiments is indicated within each bar.

DRG neuron cell bodies respond to GABA_A_ receptor agonists with sizable currents (11–15). In addition, it was recently demonstrated that nociceptive DRG neurons can produce GABA and release it from peripheral nerve terminals (9); but is there a physiological GABAergic transmission within the DRG? To start answering this question, we first tested whether DRG neuron somata can release GABA. First, rat DRG neurons were cocultured with HEK293 cells transiently cotransfected with α_1_, β_2_, and γ_2_ GABA_A_ subunits and GFP (HEK_GABAA_ cells). We then performed “sniffing” patch-clamp recordings from HEK_GABAA_ cells juxtaposed to small-diameter DRG neurons (Figure 1A). Robust inward currents were recorded from HEK_GABAA_ cells in response to 200 μM GABA (Figure 1, B–G). Application of the TRPV1 agonist capsaicin (CAP; 1 μM) produced inward currents in 7 of 10 HEK_GABAA_ cells; these currents were similar in kinetics (although smaller in amplitude) to GABA-induced currents (Figure 1, B and F). HEK_GABAA_ cells in monoculture (without DRG coculture) or nontransfected HEK cells (HEK_control_) juxtaposed to small-diameter DRG neurons in HEK_control_-DRG coculture never responded to CAP (Figure 1, C, D, and F). We were unable to elicit a response in HEK_GABAA_ cells when we mechanically stimulated juxtaposed DRG neurons of any size (Figure 1, E and G), although this could have been an issue of experimental setup. These data strongly suggest that some small-diameter, CAP-sensitive (presumed nociceptive) DRG neurons are capable of releasing GABA when stimulated.

Next, we used HPLC to detect release of GABA into the extracellular space in either dissociated DRG cultures or acutely extracted whole-DRG preparation. Depolarization of DRG cultures with elevated extracellular K^+^, CAP, bradykinin, or ATP produced a robust release of GABA into the extracellular space (Figure 1H); similar results were obtained with freshly extracted whole DRGs (Figure 1I). In the CNS GABA is generally released via classical synaptic mechanisms, but can also be released via reversal of the GABA transporter GAT1 (20). The inhibitors of synaptic transmission concanamycin A (0.5 μM) and tetanus toxin (10 μg/ml) both inhibited high-K^+^-induced GABA release almost completely (Figure 1, J and K, respectively). The GAT1 inhibitor NO711 (200 μM) produced a significant increase of tonic GABA release (Figure 1L), consistent with inhibited GABA uptake. Blocking GAT1 partially reduced (but did not abolish) high-K^+^-induced release of GABA (Figure 1L); in the presence of NO711 high K^+^ induced a statistically significant increase in extracellular GABA compared with basal levels. Thus, in combination with inhibited GABA uptake, the net effect of NO711 must result in increased extracellular GABA levels. Collectively, these data suggest that both vesicular and nonvesicular mechanisms contribute to the depolarization-induced GABA release in DRGs, with the former mechanism being predominant.

### DRG neuron somata express key components of functional GABAergic transmission.

**Figure 2:**
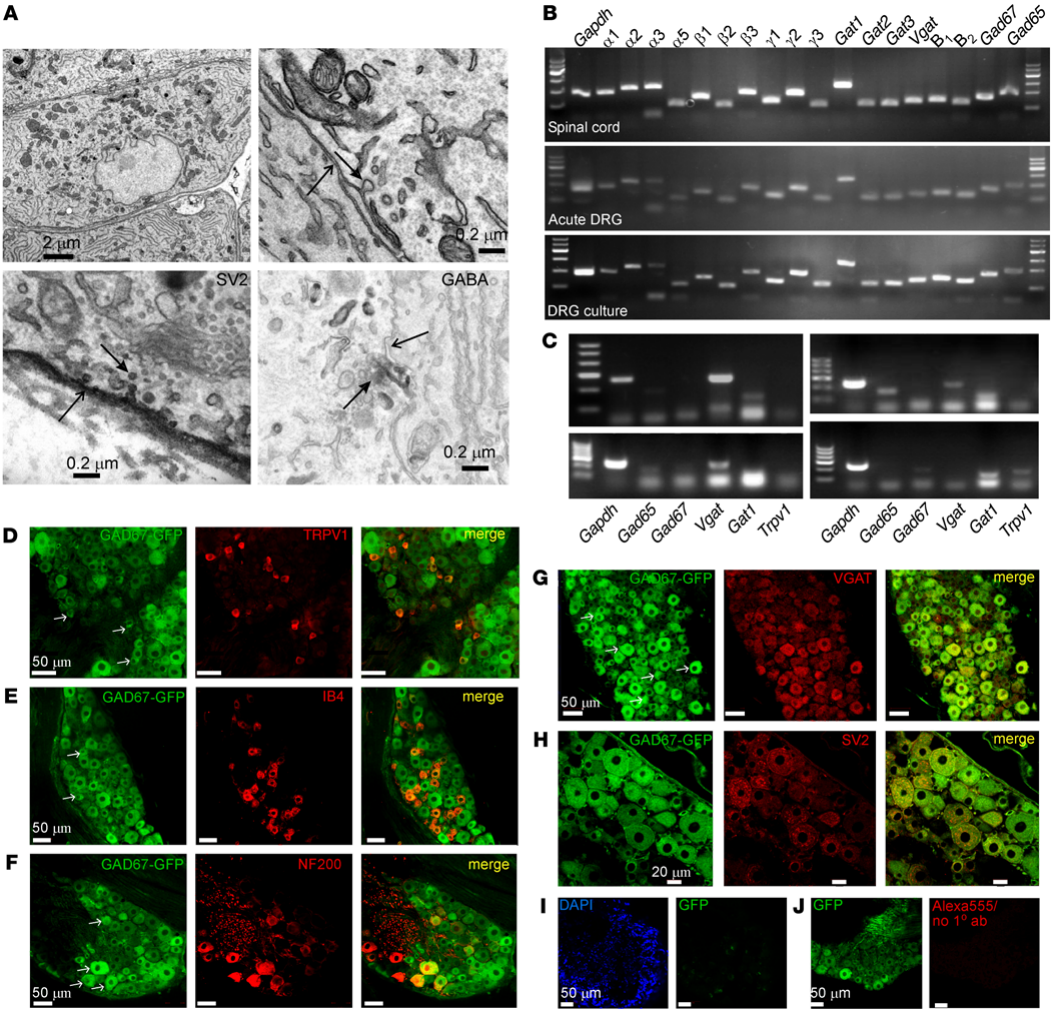
DRG neuron somata are equipped for GABAergic transmission. (**A**) Electron micrographs of DRG sections. Top panels depict examples of somatic ultrastructure revealing presence of somatic vesicles. In the top right panel a vesicle (closed arrow) can be seen apparently fusing with the membrane (open arrow). Bottom, left: SV2 immunoreactive vesicles (example indicated by closed arrow), located adjacent to and apparently fusing with the DRG neuron membrane (open arrow), which is also SV2-positive. Bottom, right: GABA immunoreactivity in vesicles (closed arrow). Open arrow indicates the DRG neuronal membrane. (**B**) Quantitative RT-PCR detection of transcripts encoding key proteins of GABAergic transmission in acutely extracted whole DRG and spinal cord tissue samples and in dissociated DRG neuron culture (48 hours), as indicated. Bands correspond to (as labeled) α_1–3_ and α_5_, β_1–3_, and γ_1–3_ subunits of GABA_A_ receptors; *Gat1–3* GABA transporters; vesicular GABA transporter *Vgat*; GABA_B_ receptors 1 and 2; and glutamate decarboxylases *Gad65* and *Gad67*. Also detected is GAPDH. (**C**) Examples of single-cell RT-PCR detection of *Gapdh*, *Gad65*, *Gad67*, *Vgat*, *Gat1*, and *Trpv1* from individual dissociated DRG neurons. Quantification is given in Supplemental Table 1. (**D**–**H**) Analysis of GAD67 expression in DRG sections from GAD67-GFP knock-in mice. Shown is the colabeling of GFP with sensory neuron markers TRPV1 (**D**), IB4 (**E**), and NF200 (**F**), as well as with VGAT (**G**) and SV2 (**H**). In **D**–**G**, arrows indicate examples of neurons expressing both GFP and the corresponding marker. (**I**) GFP labeling in WT mice. (**J**) Secondary antibody control for DRG section from GAD67-GFP knock-in mice. In **D**–**J**, micrographs within each panel are of the same magnification; scale bars are labeled on the left image in each panel.

In order to be able to release neurotransmitters, DRG neuron somata must express appropriate machinery. Electron microscopy of rat DRG sections revealed abundant presence of vesicles in DRG somata (Figure 2A); some of the vesicles expressed the synaptic vesicle marker SV2 (ref. 21; and Figure 2A, bottom left). Interestingly, some areas of somatic plasma membrane were also clearly labeled by the anti-SV2 antibody, suggesting vesicle fusion and release sites (22). Moreover, some of the vesicles were labeled with anti-GABA antibody (Figure 2A, bottom right).

While abundant expression of functional GABA_A_ and GABA_B_ receptors in DRG is well known (9, 14, 23), the presence of other components of GABAergic transmission in DRG is less well documented. Figure 2B shows the results of quantitative reverse transcription PCR (RT-PCR) detection of transcripts encoding proteins involved in GABAergic transmission from the acutely extracted whole DRG and spinal cord samples and from 48-hour DRG cultures. Multiple α, β, and γ subunits of GABA_A_ receptors were detected, with particularly strong expression of α_1–2_ and γ_1–2_ subunits, in good agreement with previous literature (14, 24). GABA_B1_ and GABA_B2_ subunits of GABA_B_ receptors were also expressed. We also detected the following transcripts: (a) the glutamate decarboxylase isoforms *Gad65* and *Gad67* (which decarboxylate glutamate to produce GABA); (b) the vesicular GABA transporter *Vgat* (which packs GABA into vesicles); and (c) the plasma membrane GABA transporters *Gat1–3* (which remove released GABA from the extracellular space). *Gad65* and *Gad67*, *Gat1–3*, and *Vgat* were detected in the acute DRG at somewhat lower but comparable levels relative to those found in the spinal cord. Among GAT subunits, GAT1 showed the strongest expression.

Since whole-DRG tissue or cultures contain non-neuronal cells, we tested whether we could detect expression of GABA-related transcripts in the individual neurons using single-cell RT-PCR. Indeed that was the case; *Gad65*, *Gad67*, and *Vgat* mRNA was detected in approximately 8%–9% of neurons, while *Gat1* was detected in a larger subpopulation (48%) of DRG neurons (Figure 2C and Supplemental Table 1; supplemental material available online with this article; https://doi.org/10.1172/JCI86812DS1). It has to be pointed out that, because of the high rate of false negatives in single-cell RNA analyses (25), the proportions of cells identified as positive for a given transcript in Supplemental Table 1 are likely underestimated.

We then used GAD67-GFP knock-in mice (26) and immunohistochemistry to characterize the GABA-producing DRG neurons. We found cytosolic expression of the reporter (GFP) in many DRG neurons of all sizes, although expression in larger-diameter neurons was more frequent (Figure 2, D–H). There was partial overlap between expression of GFP and presence of such markers of sensory neuron subtypes as TRPV1 (polymodal nociceptors; Figure 2D), IB4 (nonpeptidergic nociceptors; Figure 2E), and more so with neurofilament 200 (NF200, myelinated fibers; Figure 2F). There was a very good correlation between GAD67 (GFP) expression and that of VGAT (Figure 2G). Finally, DRG neurons were abundantly labeled by anti-SV2 antibody with a characteristic punctate staining suggesting vesicular localization of SV2 (Figure 2H); most SV2-positive neurons also expressed GAD67.

**Figure 3:**
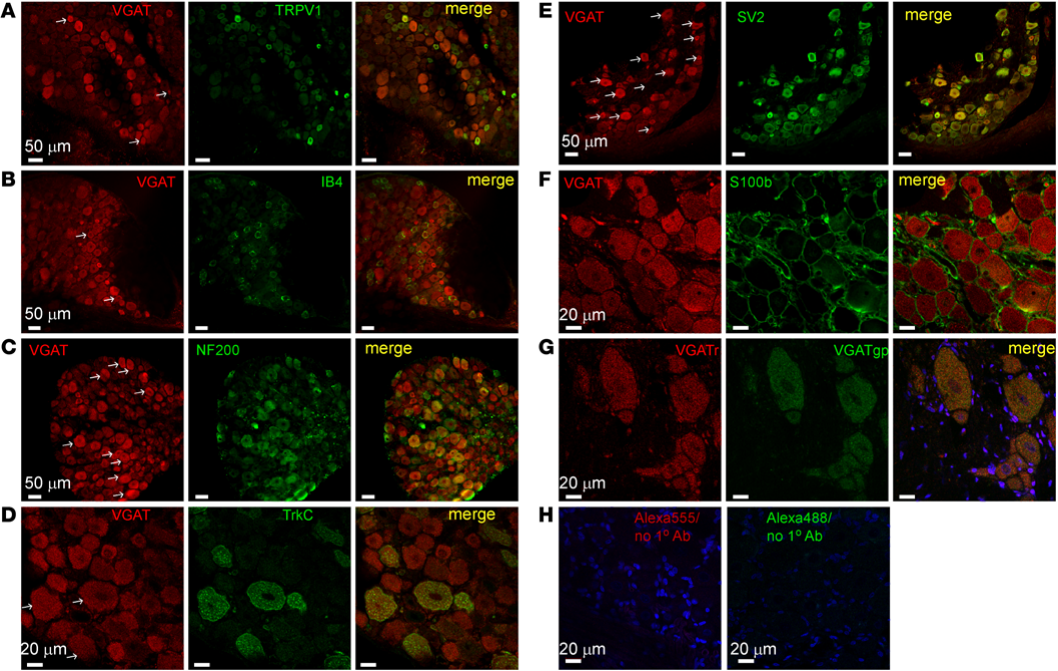
Expression of VGAT in rat DRG. (**A**–**G**) Shown is the colabeling of VGAT with sensory neuron markers TRPV1 (**A**), IB4 (**B**), NF200 (**C**), and TrkC (**D**) as well as with SV2 (**E**) and satellite glia marker S100b (**F**). In **A**–**E**, arrows indicate examples of neurons expressing both VGAT and the corresponding marker. (**G**) Costaining of the same DRG section with 2 different antibodies raised in rabbit (VGATr) and guinea pig (VGATgp). (**H**) Control for secondary antibodies used. Micrographs within each panel are of the same magnification; scale bars are labeled on the left image in each panel. Somatic diameter distribution of VGAT-positive DRG neurons is given in Supplemental Table 2.

We also analyzed expression of VGAT in rat DRG (Figure 3 and Supplemental Table 2). Similarly to GFP in GAD67-GFP knock-in mice, VGAT expression was found in neurons of all sizes but was twice as frequent in larger-diameter neurons as compared with small ones (Supplemental Table 2). However, since there are more small-diameter neurons than larger ones in DRG (27), the total number of VGAT-positive small and large neurons was comparable (Supplemental Table 2). Again, VGAT immunofluorescence was found in some TRPV1- and IB4-positive neurons (Figure 3, A and B) but was more often found in larger, NF200- and TrkC-positive neurons (Figure 3, C and D). There was again a good colocalization of VGAT with SV2 (Figure 3E). Importantly, VGAT immunofluorescence was absent in satellite glia (as confirmed with the glial marker S100b; Figure 3F). Two distinct VGAT antibodies stained the exact same subpopulation of neurons (Figure 3G), and omission of primary antibodies resulted in almost complete absence of immunofluorescence (Figure 3H), indicating that staining was specific.

### Stimulation of GABA system in DRG reduces acute pain transduction in vivo.

**Figure 4:**
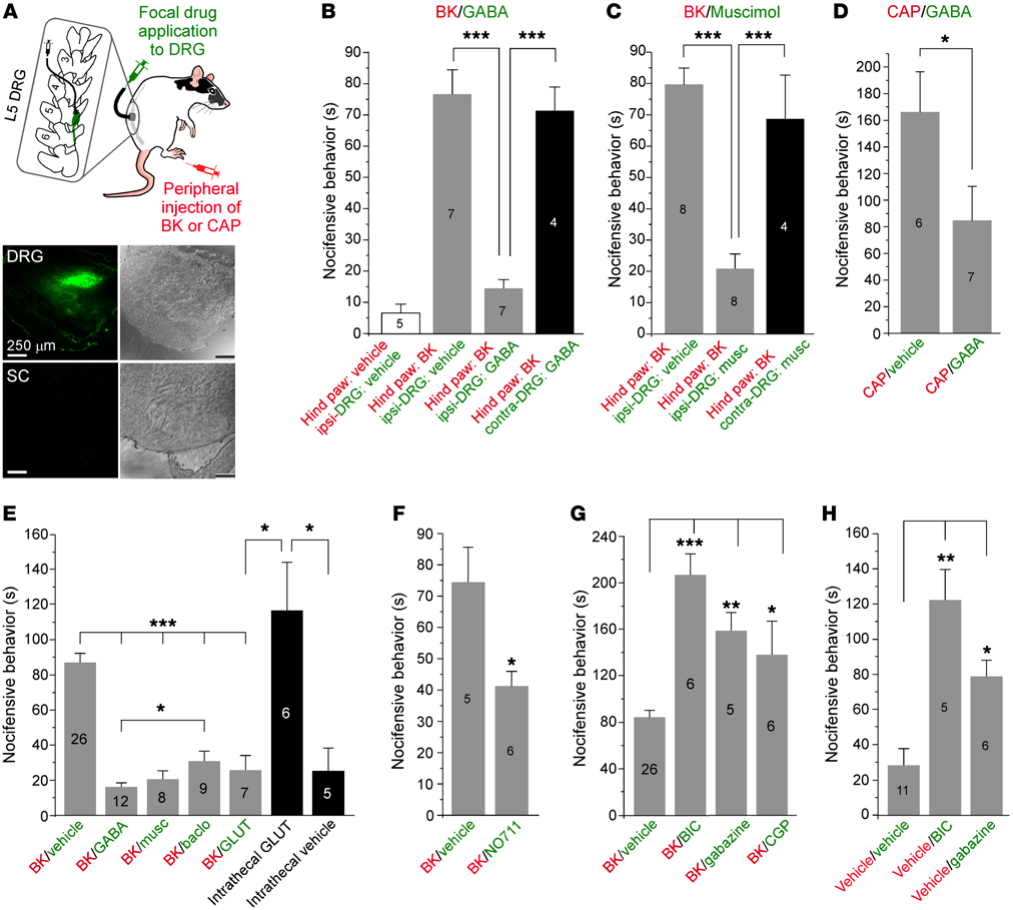
Focal application of GABA to DRG reduces pain in vivo. (**A**) DRG cannula implant. Top inset shows schematic of the procedure; colors indicate route of drug administration. Images on the bottom are bright-field (left) and fluorescent (right) micrographs of DRG (top) and proximal spinal cord (bottom) after the focal application of a fluorescent dye, CFSE (20 μM in 5 ml), via the DRG cannula. All images were taken at the same magnification (as indicated by the scale bars). (**B**) Focal DRG application of GABA (200 μM, 5 μl) via DRG cannula strongly reduced pain produced by hind-paw injection of bradykinin (BK, 200 μM, 50 μl). (**C**) Similar to **B** but the specific GABA_A_ receptor agonist muscimol (musc, 200 μM, 5 μl) was applied. (**D**) Focal DRG application of GABA (200 μM, 5 μl) via DRG cannula reduced pain produced by hind-paw injection of capsaicin (CAP, 20 μM, 50 μl). (**E**) Comparison of effects of focal DRG application of GABA, muscimol, baclofen, and glutamate (GLUT) (all at 200 μM, 5 μl) on the BK-induced nocifensive behavior. Black bars depict effect of intrathecal injection of glutamate (200 μM, 10 μl) and a vehicle control. (**F**) Focal DRG application of GAT1 inhibitor NO711 (200 μM, 5 μl) significantly reduced pain produced by hind-paw injection of BK. (**G**) Focal DRG application of GABA_A_ receptor inhibitors bicuculline (BIC, 200 μM, 5 μl) and gabazine (200 μM, 5 μl) and GABA_B_ receptor inhibitor CGP35348 (CGP, 200 μM, 5 μl) exacerbated nocifensive behavior produced by hind-paw BK injection. (**H**) In the absence of plantar injection of BK, focal DRG application of bicuculline and gabazine resulted in distress in the paw and nocifensive behavior similar to that induced by plantar injection of BK. In **B**–**H** the number of experiments is indicated within or above each bar; asterisks indicate significant difference from the appropriate control: *P < 0.05, **P < 0.01, ***P < 0.001 (Kruskal-Wallis ANOVA with Mann-Whitney test for between-group comparison).

Experiments reported hitherto established that DRG neurons can produce and release GABA, but is there a physiological role for such somatic/perisomatic release? We tested the effect of direct focal GABA infusion into the DRG on pain-related behavior in rats (see Methods). In this approach, a cannula that allows delivery of small volumes of solutions directly to the selected spinal ganglion is inserted into a hole drilled through the transverse process of the corresponding vertebra (ref. 28 and Figure 4A). Since exogenous GABA would have strong inhibitory action on nociceptive transmission in the spinal cord, we first verified that this injection technique delivers drugs specifically to the DRG. We injected a fluorescent dye, CFSE (20 μM; 5 μl), through the cannula and tested the labeling of DRG and the proximal spinal cord. Confocal fluorescent imaging revealed a complete lack of staining in the spinal cord, while there was abundant fluorescence in the DRG (Figure 4A, bottom, and Supplemental Figure 1). We then investigated how focal DRG application of drugs targeting the GABA system would affect nociceptive transmission.

In the first series of experiments we induced painful sensation in a hind paw of a rat by a plantar injection of the noxious inflammatory peptide bradykinin (BK) or the TRPV1 agonist capsaicin (CAP) and tested the effect of focal DRG preapplication of GABA receptor agonists and antagonists, or a GABA uptake blocker, on the BK/CAP-induced nocifensive behavior (licking, biting, or flinching the paw). Focal application of GABA (200 μM; 5 μl; Figure 4B) or the GABA_A_ agonist muscimol (200 μM; 5 μl; Figure 4C) to the DRG strongly suppressed nocifensive behavior induced by the hind-paw BK injection (200 μM; 50 μl). Focal DRG application of saline produced no effect. When BK was injected to the paw contralateral to the cannula implant, neither GABA nor muscimol reduced the BK-induced nocifensive behavior (Figure 4, B and C). Absence of contralateral effects provides additional evidence against a spillover from the site of DRG injection to the spinal cord. Nocifensive behavior induced by hind-paw injection of CAP (20 μM; 50 μl) was also significantly reduced by the DRG application of GABA (Figure 4D). Focal DRG infusion of the GABA_B_ receptor agonist baclofen (200 μM; 5 μl; Figure 4E) also produced marked reduction of the BK-induced nocifensive behavior, although the effect was significantly less pronounced than that of GABA.

DRG neurons use glutamate as the main excitatory neurotransmitter at their central terminals, but these neurons also express a variety of glutamate receptors (8); thus we tested the effect of focal DRG application of glutamate (200 μM; 5 μl). Strikingly, glutamate infusion also strongly attenuated BK-induced pain (Figure 4E). However, when the same amount of glutamate was injected intrathecally (such injections would affect both spinal cord and DRG), it produced strong bilateral nocifensive behavior (flinching and biting the hind paws) even without hind-paw injection of a proalgesic compound (Figure 4E). These clearly opposite effects of glutamate applied to DRG (analgesia) or spinal cord/DRG (nociception) suggested that glutamate applied via the DRG cannula does not reach the spinal cord. A likely explanation for the unexpected inhibitory action of glutamate in DRG will be discussed along with the computer modeling data below.

Focal DRG application of the GAT1 inhibitor NO711 (200 μM; 5 μl; Figure 4F) also significantly attenuated the BK-induced nocifensive behavior, suggesting a role of endogenous GABA in nociceptive transmission; the result is also in agreement with an increase of tonic DRG GABA levels induced by NO711 (Figure 1H). Another intriguing finding was that focal application of the GABA_A_ antagonists bicuculline (200 μM; 5 μl) and gabazine (200 μM; 5 μl) and, to a lesser extent, the GABA_B_ receptor antagonist CGP35348 (200 μM; 5 μl) exacerbated pain produced by the plantar injection of BK (Figure 4G). Even without BK injection, focally applied bicuculline and gabazine generated obvious flinching and biting of the ipsilateral paw (Figure 4H), suggesting that there may indeed be tonic GABA-mediated inhibition within the DRG that, when removed, results in spurious peripheral nerve activity.

We also analyzed concentration dependence of the behavioral effects of GABA, muscimol, bicuculline, and NO711 (Supplemental Figure 2, A–E). All the compounds displayed concentration dependence within the used concentration range; prominent analgesic effects of GABA and muscimol were evident at 20 μM.

Since rat hind paws are innervated by L4 as well as L5 DRGs, we also tested the effect of GABA applied through the cannula implanted to the L4 DRG (Supplemental Figure 2F). Focal application of GABA to the L4 DRG also reduced BK-induced nocifensive behavior but was less efficacious as compared with the L5 injections. To further confirm the accuracy of DRG cannula injections, we measured GABA, NO711, and bicuculline levels in DRG and proximal spinal cord after the L5 DRG cannula injection using HPLC. The drugs were detected in the DRG but not in the spinal cord (Supplemental Figure 3).

Focal injection of GABA significantly decreased sensitivity of rats to thermal and mechanical stimuli as measured with the Hargreaves and von Frey tests. Thus, hind-paw withdrawal latency upon presentation with radial heat (Supplemental Figure 4A) and the threshold for sensitivity to mechanical stimulation (Supplemental Figure 4B) were both increased. Bicuculline significantly increased sensitivity to both types of stimulation (Supplemental Figure 4, C and D).

Plantar paw injection of bicuculline (200 μM; 50 μl; Supplemental Figure 4E) was largely without an effect. Likewise, plantar injection of GABA (200 μM; 50 μl) did not produce noticeable acute effects (Supplemental Figure 4F) and failed to reduce BK-induced nocifensive behavior (Supplemental Figure 4G). The experiments with focal application of drugs to the spinal sensory ganglia in vivo established that, acting within the ganglion, exogenous or endogenous GABA can strongly suppress nociceptive transmission.

Since either endogenous release or exogenous GABA delivery to DRG reduced peripheral pain responses, we tested whether GABA application to DRG neurons reduces synaptic input to the spinal cord in vivo. We quantified the effect of focal DRG application of GABA via the DRG cannula on the activation of spinal postsynaptic neurons by the hind-paw injection of CAP using a neuronal activation marker, c-Fos (29). No nuclear c-Fos staining was seen in the spinal cord of naive animals (Supplemental Figure 5A). Hind-paw injection of CAP (20 μM; 50 μl) induced robust nuclear c-Fos staining in dorsal horn ipsilateral to the injection site, especially in superficial laminae I–II (Supplemental Figure 5B); no contralateral staining was apparent (Supplemental Figure 5C). DRG application of GABA significantly reduced CAP-induced c-Fos expression in the ipsilateral dorsal horn (Supplemental Figure 5, A–F), suggesting that it indeed reduced excitatory input to the spinal cord.

### Stimulation of GABA system in DRG alleviates chronic neuropathic and inflammatory pain.

**Figure 5:**
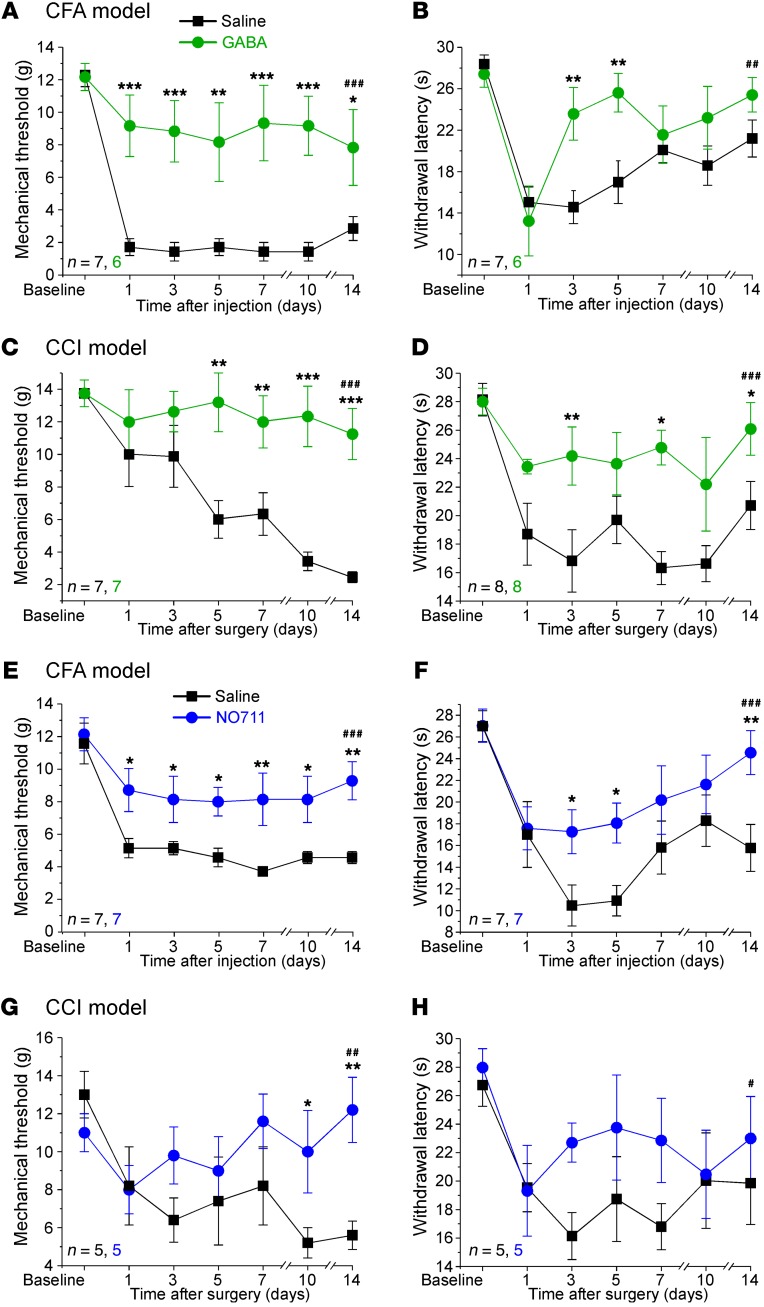
Focal infusion of GABA or GABA reuptake inhibitor to DRG alleviates chronic pain in vivo. (**A** and **B**) Focal DRG application of GABA (200 μM, ~0.5 μl/h) via osmotic minipump significantly alleviated mechanical (**A**) and thermal (**B**) hyperalgesia produced by the plantar injection of CFA. (**C** and **D**) Similar to **A** and **B** but a chronic constriction injury (CCI) was applied to a sciatic nerve instead of CFA injection. (**E**–**H**) Similar to **A** and **B** but GAT1 inhibitor NO711 (200 μM, ~0.5 μl/h) was applied via the minipump implant instead of GABA. In **A**–**H**, the CFA/CCI procedure and the minipump implantation were performed at the same time. Number symbols indicate significant difference between saline and drug data sets: ^#^*P* < 0.05, ^##^*P* < 0.01, ^###^*P* < 0.001; asterisks indicate the difference between groups within the corresponding time point: *P < 0.05, **P < 0.01, ***P < 0.001 (2-way ANOVA with Bonferroni correction for saline vs. drug comparison or 1-way ANOVA with Fisher LSD post hoc test for comparison between the groups). Number of experiments is indicated as *n* in each panel.

We next tested whether targeting the GABAergic system in DRG can alleviate chronic pain. We used a chronic constriction injury (CCI) model of neuropathic pain and peripheral injection of CFA as a model of chronic inflammatory pain (see Methods); both models are widely used in pain research (30). In order to achieve prolonged local release of GABA or GABA mimetics into the DRG, we implanted osmotic minipumps in a manner similar to the DRG cannula implantation described above (see Methods, Supplemental Figure 6A). The pump slowly releases its content locally at the site of implantation at a speed of approximately 0.5 μl/h for approximately 14 days. In most experiments we installed minipumps filled either with saline or with a GABA modulator at the time of CCI surgery or CFA injection and monitored animals’ sensitivity to the mechanical and thermal stimulation for the next 14 days. As expected, in both CCI and CFA models a robust mechanical and thermal hyperalgesia was observed in rats receiving saline (Figure 5 and Supplemental Figure 6); there was slight spontaneous recovery of the thermal hyperalgesia over the last week of observation in the CFA model. Yet animals that were infused with GABA (Figure 5, A–D) or muscimol (Supplemental Figure 6, C–F) or with the GAT1 inhibitor NO711 (Figure 5, E–H) were significantly less susceptible to both mechanical and thermal hyperalgesia in either of the models. Moreover, when a GABA-filled osmotic minipump was implanted on day 8 after the CCI injury, such a procedure rapidly and significantly attenuated mechanical hyperalgesia induced by the CCI (Supplemental Figure 6B). These results suggest that not only acute but also chronic pain can be attenuated by targeting of the GABA system in DRG; moreover, alleviation of chronic pain with NO711 suggests that endogenous GABA production/release mechanisms in DRG can be targeted for pain relief.

### Chemo- or optogenetic depolarization of GABAergic DRG neurons reduces pain transduction.

**Figure 6:**
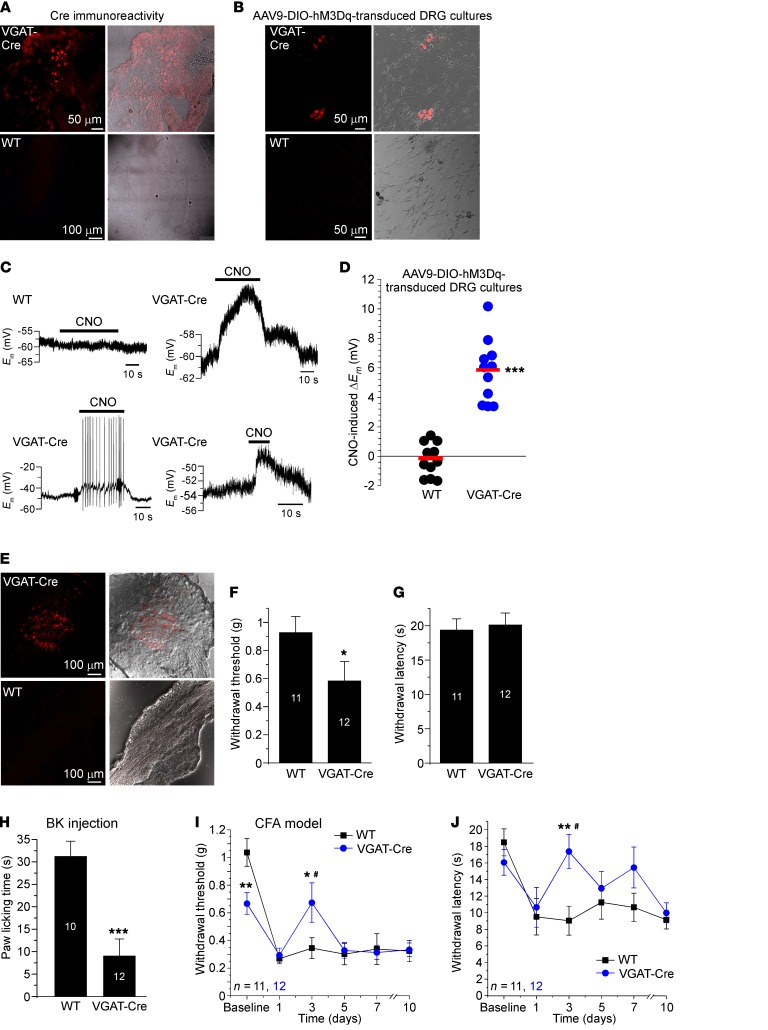
Chemogenetic depolarization of DRG neurons via designer Gq receptors reduces peripherally induced pain. (**A**) Immunohistochemical detection of Cre in DRG section from the VGAT-Cre mice. (**B**) Expression of mCherry-tagged hM3Gq in cultured DRG neurons from the VGAT-Cre mice (and WT littermates) transduced with AAV9-DIO-hM3Dq DREADD virus. (**C**) Examples of recordings from hM3Dq-expressing DRG neurons depolarized by the application of 10 μM clozapine-*N*-oxide (CNO). (**D**) A scatter plot summary of experiments shown in **C**. ***Significant difference from the baseline (paired, 2-tailed *t* test). (**E**) Expression of hM3Dq in L4 DRG of the VGAT-Cre mouse in vivo transduced with the AAV9-DIO-hM3Dq virus. (**F** and **G**) Effect of the i.p. injection of CNO (0.9% saline, 5 mg/kg) on the mechanical (**F**) and thermal (**G**) sensitivity of VGAT-Cre mice injected with hM3Dq DREADD viruses into L4 DRG (compared with virus-injected WT littermates). (**H**) CNO injection (i.p.) reduced nocifensive behavior (paw licking) of VGAT-Cre mice injected with hM3Dq DREADD viruses into L4 DRG (compared with virus-injected WT littermates). In **F**–**H**, asterisks indicate significant difference from the WT mice: *P < 0.05, ***P < 0.001 (unpaired, 2-tailed *t* test); number of experiments is shown within the bars. (**I** and **J**) CNO injection (i.p.) reduced mechanical (**I**) and thermal (**J**) hyperalgesia in CFA model of inflammatory pain in VGAT-Cre mice injected with hM3Dq DREADD viruses into L4 DRG (compared with virus-injected WT littermates). ^#^Significant difference between VGAT-Cre and WT mice data sets. Asterisks indicate the difference between groups within the corresponding time point: *P < 0.05, **P < 0.01 (2-way ANOVA with Bonferroni correction for VGAT-Cre vs. WT comparison or 1-way ANOVA with Fisher LSD post hoc test for comparison between the groups). The number of experiments is indicated as *n* in each panel.

Since DRG neurons express VGAT (Figures 2 And 3 and ref. 9), we used VGAT-IRES-Cre mice (31) to develop a chemogenetic approach for specific depolarization of GABAergic DRG neurons in vivo. In this mouse line Cre recombinase expression is directed to the VGAT-expressing neurons, without disrupting endogenous VGAT expression (31). DRG sections from the VGAT-Cre mice expressed Cre (Figure 6A), and a population of cultured DRG neurons (but not glia) from VGAT-Cre mice was successfully transduced with AAV9 viruses coding for the mCherry-tagged FLEXed DREADD (designer receptor exclusively activated by designer drug; ref. 32) receptors coupled to Gq (hM3Gq; see Methods). DRG cultures from the WT littermates were not transduced (Figure 6B). Application of the DREADD ligand clozapine-*N*-oxide (CNO) to DREADD-expressing DRG neurons reliably depolarized these by approximately 4–10 mV while having no effect on the virus-treated neurons from WT mice (Figure 6, C and D). In 2 of 11 neurons CNO induced AP firing (see example in Figure 6C, bottom left), although the majority of neurons did not fire.

Next, we injected Gq DREADD viruses into L4 DRG of VGAT-Cre mice (as well as the WT littermates) to test whether CNO-induced depolarization of VGAT-expressing (GABAergic) DRG neurons would affect nociceptive transmission in vivo. L4 DRG was chosen because, while in rats L4, L5, and L6 spinal nerves are major contributors to the sciatic nerve, in mice the contributing nerves are L3, L4, and L5 (33). mCherry fluorescence was readily detectable in DRG of virus-injected (see Methods) VGAT-Cre mice but not WT mice (Figure 6E). Injection of CNO (5 mg/kg i.p., 30 minutes before the test) slightly increased basal mechanical (but not thermal) sensitivity of VGAT-Cre mice (Figure 6, F and G), as is expected from the depolarization of peripheral nerve terminals. Importantly, CNO preinjection (30 minutes) strongly suppressed nocifensive behavior induced by the plantar injection of BK (Figure 6H), suggesting that depolarization of GABAergic neurons is indeed antinociceptive. Furthermore, CNO injections significantly reduced both mechanical (Figure 6I) and thermal (Figure 6J) hyperalgesia in the CFA model of inflammatory pain. In these experiments CNO was repeatedly injected 30 minutes before each measurement. The antihyperalgesic effect of CNO peaked at the third day after CFA injection, after which point hyperalgesia recovered; perhaps this suggests gradual desensitization of the DREADD system in vivo.

In our DREADD experiments we were unable to deliver CNO to DRG directly, since the virus injection procedure required removal of both spinous and transverse processes of L4 DRG (see Methods) and, therefore, a cannula or minipump could not be implanted. In order to deliver a depolarizing stimulus to DRG with high precision, we used VGAT-ChR2-EYFP mice (34), in which all VGAT-expressing neurons are expected to express channelrhodopsin-2. Consistent with VGAT immunohistochemistry (Figure 2) and the VGAT-Cre mouse experiments (Figure 6), a population of DRG neurons of various sizes expressed yellow fluorescent protein (YFP) (Supplemental Figure 7A). We then used the sniffing patch approach to test whether optical stimulation of DRG neurons dissociated from DRG of VGAT-ChR2-EYFP mice can trigger GABA release. Blue light (473 nm) stimulation of such DRG neurons cocultured with HEK_GABAA_ cells induced detectable inward currents in 11 of 24 HEK_GABAA_ cells juxtaposed to DRG neuron somata (Supplemental Figure 7, B and C). HEK_GABAA_ cells in monoculture did not respond to blue light (Supplemental Figure 7, B and C). HEK_GABAA_ cells cocultured with DRG neurons from the WT littermate control mice were also largely unresponsive to blue light, although in 4 of 18 neurons very small inward currents could be detected (though it was not statistically significant); these may have been produced by GABA released from heat-sensitive DRG neurons.

Next, we used VGAT-ChR2-EYFP mice with an optical fiber implanted through a cannula guide in a manner similar to the DRG cannula or minipump implants (see Methods and Supplemental Figure 7D); in this way DRG can be stimulated optically in vivo in freely moving mice with high spatial precision. First, we tested the effect of optical stimulation on the pain-related (nocifensive) behavior produced by plantar BK injection. Ipsilateral stimulation by light significantly reduced BK-induced paw licking in VGAT-ChR2-EYFP mice as compared with WT littermates (Supplemental Figure 7E); without optical stimulation there was no difference in BK-induced nocifensive behavior between the WT and transgenic animals (Supplemental Figure 7F). Importantly, when BK was injected to the contralateral paw, optical stimulation no longer affected BK-induced pain responses (Supplemental Figure 7G), verifying the precision of optical stimulation. Optogenetic stimulation of ChR2-expressing DRG neurons is likely to induce not only GABA but also glutamate release from these neurons. If released from central terminals in the spinal cord, glutamate is expected to produce excitatory/painful effects (similarly to the intrathecal glutamate injection; Figure 4E). Yet optogenetic stimulation of DRG in VGAT-ChR2-EYFP mice was clearly analgesic (as was the DRG-applied glutamate; Figure 4E). Thus, regardless of the transmitter, the antinociceptive effect of optogenetic DRG stimulation is likely to be mediated by mechanisms localized to the somatic/perisomatic compartments of the afferent fibers (see also the results of the computer modeling presented below).

### GABA_A_ activation impedes propagation of APs through DRG: an electrophysiological and computational study.

**Figure 7:**
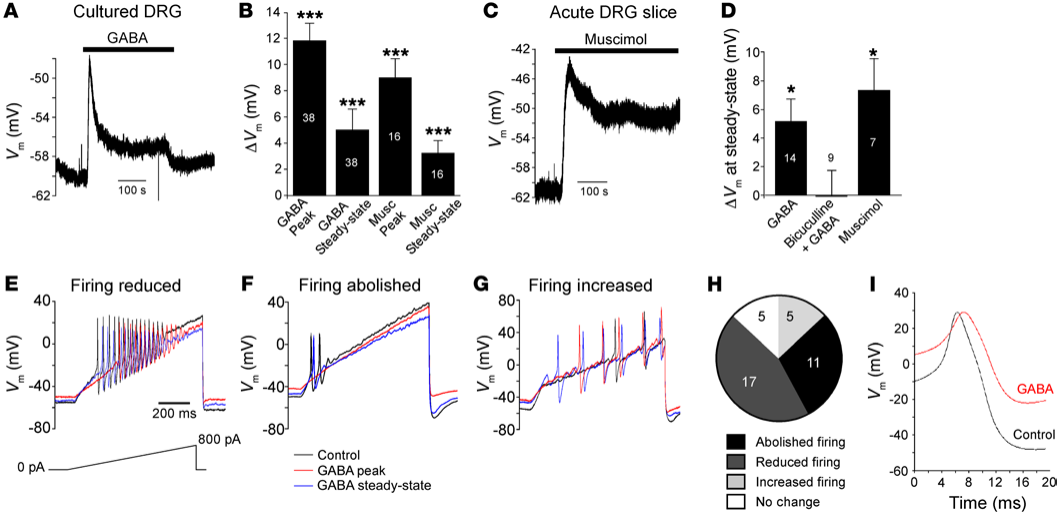
Effects of GABA on the somatic excitability of DRG neurons. GABA_A_ agonists induce depolarization of DRG neurons in culture (**A** and **B**) and in acute slice preparation (**C** and **D**). Top traces depict exemplary current-clamp recordings during application of 200 μM GABA or 10 μM muscimol (as indicated by horizontal bars). (**B**) Summary of effects of GABA and muscimol during peak and plateau (steady-state) phases of response recorded from cultured small-diameter DRG neurons. (**D**) Summary of a steady-state depolarization produced by GABA and muscimol in DRG slices and also the inhibition of GABA response by bicuculline; number of recordings is indicated within the bars. Asterisks indicate significant difference from the baseline: *P < 0.05, ***P < 0.001 (paired, 2-tailed *t* test). (**E**–**G**) GABA inhibits AP firing in most DRG neurons. Shown are exemplary voltage traces before GABA application (black) and also during the peak (red) and steady-state (blue) response. Examples represent neurons in which AP firing is reduced (**E**), abolished (**F**), or increased (**G**). Stimulus protocol (current ramp) is depicted beneath the traces in **E**. (**H**) Proportion of neurons in which AP firing was abolished, reduced, or increased or in which there was no response. (**I**) GABA reduces AP amplitude; further quantification of the effects of GABA on DRG excitability is provided in Supplemental Tables 3 and 4.

In contrast to adult CNS, where GABA_A_ receptors hyperpolarize neurons (35), in somatosensory neurons GABA_A_ (or other Cl^–^ channels) induces depolarization (7, 12, 19, 36, 37) due to the relatively high intracellular Cl^–^ concentrations (38). Accordingly, GABA (200 μM) or the specific GABA_A_ receptor agonist muscimol (10 μM) produced robust depolarization in the majority of cultured small-diameter DRG neurons (Figure 7, A and B). Likewise, in intracellular sharp electrode recordings from acute DRG slices, GABA and muscimol produced comparable responses (Figure 7, C and D; note that steady-state current amplitude is plotted in Figure 7D). Bicuculline (50 μM) completely abolished GABA-induced depolarization (Figure 7D), confirming that it is mediated by GABA_A_ receptors. Similar responses in both cultured DRG neurons and neurons in acute DRG slices suggest that a high density of functional GABA_A_ receptors in DRG neuron somata reflects their native phenotype.

Although neuronal membrane depolarization is usually associated with increased excitability, a large GABA_A_ conductance can also produce a shunting effect preventing AP generation (39). We investigated the effects of GABA on AP firing in small-diameter (presumed nociceptive) cultured DRG neurons using the gramicidin-perforated patch clamp (gramicidin pores are impermeable to Cl^–^ [ref. 40]; therefore endogenous Cl^–^ concentration is preserved). In the majority of neurons, firing in response to slow depolarizing current ramps was reduced (17 of 38; Figure 7, E and H) or abolished (11 of 38; Figure 7, F and H) by GABA. In a small proportion of neurons GABA either induced no effect (5 of 38; not shown) or slightly increased firing (5 of 38; Figure 7, G and H). In total, 33 of 38 neurons (87%) responded to GABA with changes in excitability; detailed analysis of these effects is presented in Supplemental Tables 3 and 4. In all but 5 neurons that showed no response, GABA induced significant depolarization and also reduced the first AP amplitude (Figure 7I). In neurons where firing was reduced there was a significant increase in the threshold current (Supplemental Table 3).

**Figure 8:**
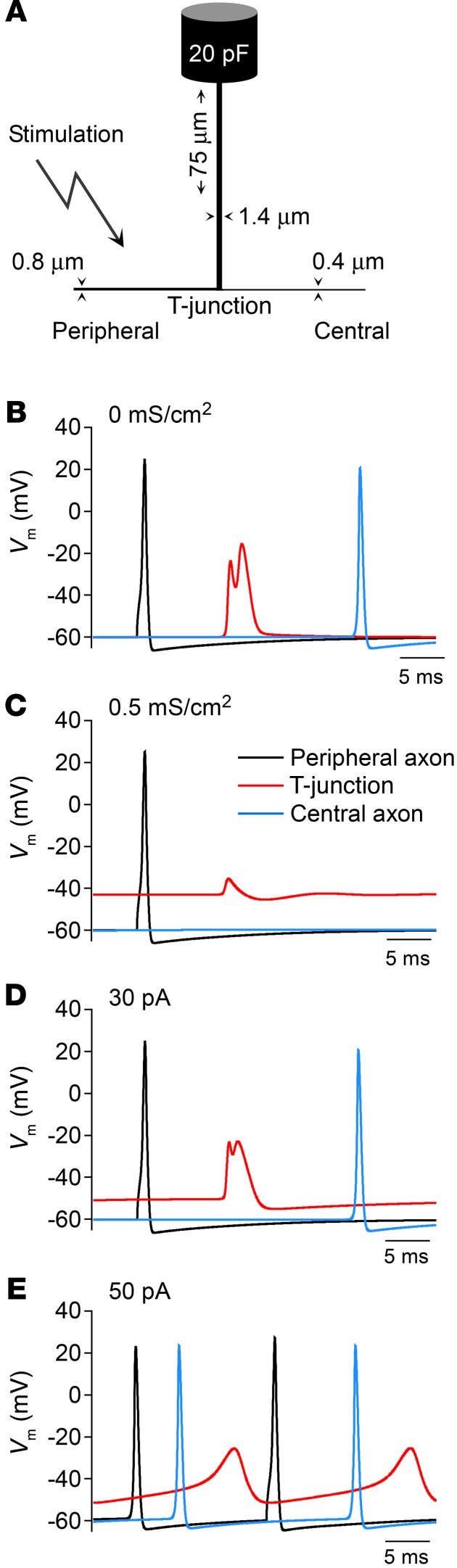
Activation of somatic GABA_A_ conductance results in failure of peripheral AP at axonal bifurcation in a mammalian nociceptive neuron model. (**A**) Schematic illustration of a model (not to scale). (**B**) In the absence of GABA_A_ current spikes propagated through the T-junction to the central axon. (**C**) Activation of a membrane conductance (*E*_rev_ = –40 mV) within the DRG depolarized the T-junction and blocked spike propagation. (**D** and **E**) Effect of somatic depolarization without an increase in membrane conductance. Somatic depolarizing current injection of 30 pA depolarized the T-junction while having no effect on spike propagation (**D**). Greater depolarizing stimulus triggered spontaneous firing (**E**).

Taken together, electrophysiological recordings suggest that, despite depolarization, the overall effect of GABA on somatic excitability of DRG neurons is inhibitory. However, because of specific morphology of DRG neurons, somatic excitability is not necessary for the AP propagation from the periphery to the spinal cord (2). So how might GABAergic modulation of somatic/perisomatic compartments of DRG neurons affect the AP propagation through the ganglion? Both measurements (28, 41–44) and simulations (28, 45, 46) suggest that the axonal bifurcation (T-junction; Figure 8A) within sensory ganglia influences the transmission of passing spikes serving a filtering function. Here we constructed a biophysical model of the ganglionic portion of an adult, mammalian nociceptive neuron using available morphology from the literature (refs. 47–50 and Figure 8A) to test the effect of somatic GABA-like conductance on propagation of spikes through DRG. Activation of a membrane conductance with reversal potential at –40 mV, corresponding to the Cl^–^ equilibrium potential of nociceptive neurons (38), depolarized membrane potential (E_Cl_) at the T-junction, resulting in AP failure (Figure 8, B and C). Similar somatic depolarization without an increase in membrane conductance had no effect on spike propagation (Figure 8D), while greater depolarization was more likely to generate spontaneous firing (Figure 8E). The combination of Na^+^ channel inactivation and increased membrane conductance (GABA_A_ current), along with the low safety factor for spike conduction through the T-junction (28, 46), contributed to blocking spike propagation through the DRG. Consistent with electrophysiological data (Figure 7, E–H, and Supplemental Tables 3 and 4), under certain conditions (e.g., varying *E*_Cl_, threshold voltage, or GABA_A_ receptor density) GABA_A_ receptor activation triggered AP firing (not shown). Yet the main conclusion of the modeling was that over a physiologically relevant range of parameters the activation of large ganglionic GABA_A_ currents is likely to block AP propagation through the T-junction in DRG. It is worth pointing out that GABA-induced depolarization may still produce excitatory and proalgesic effects at the peripheral nerve terminals or elsewhere along the axons, as these sites have a higher AP safety factor as compared with T-junction. Indeed, increased peripheral depolarization mediated by GABA_A_ receptors was suggested to contribute to inflammatory pain (7, 37).

Our model also provides a straightforward explanation for “paradoxical” effects of glutamate receptors in DRG: injection of glutamate through the DRG cannula in vivo resulted in antinociceptive effect (Figure 4E, but compare the intrathecal injection of glutamate). How does a “classic” excitatory neurotransmitter inhibit peripheral neuron excitability? Our data and modeling suggest that large depolarizing currents produced by the ionotropic glutamate receptors at the somatic/perisomatic level could, similarly to depolarizing GABA_A_ currents, result in AP failure to propagate past the T-junction. Yet, applied to the postsynaptic neurons in the spinal cord (i.e., via the intrathecal injection), glutamate exerts the expected excitation. The modeling results also align well with the antinociceptive effects of chemo- or optogenetic stimulation of GABAergic DRG neurons in vivo (Figure 6 and Supplemental Figure 7) and with the recent finding that sensory neuron–specific knockout of the NR1 subunit of NMDA receptors resulted in unexpected hyperexcitability and pain hypersensitivity (8).

## Discussion

Here we identified fully functional local GABAergic communication between DRG neurons. We demonstrated that key components of the GABAergic transmission (GABA_A_ receptors, transporters, and synthesis enzyme) are expressed in DRG and are functional. Depolarizing stimuli induced GABA release in DRG, and, in turn, most DRG neurons responded to GABA with reduced excitability. Focal application of GABA, GABA_A_ agonists, and GABA reuptake inhibitors to DRG as well as targeted chemo- or optogenetic stimulation of GABAergic DRG neurons in mice produced strong analgesic effects in vivo. Most importantly, targeting the somatic/perisomatic GABAergic system in DRG is efficacious in alleviating not only acute but also chronic pain.

Our data suggest that GABA can be produced by several subtypes of DRG neurons. While this provides a framework for multiple integratory mechanisms, our data support the following conclusions as the minimum: (a) subpopulations of large-, medium-, and small-diameter DRG neurons can release GABA upon stimulation; and (b) almost all small-diameter DRG neurons can respond to GABA. The released GABA is removed from the extracellular space by satellite glia, as reported previously (51, 52), and probably by neurons as well. Glial GABA release (perhaps nonvesicular, since glia lack VGAT) may also contribute to signaling in DRG, particularly to setting tonic GABA levels (53, 54). Of particular importance is the fact that not only GABA, but also the GABA reuptake inhibitor NO711, produced antinociceptive effect when locally delivered to DRG in vivo. Moreover, GABA_A_ receptor antagonists, delivered in the same way, not only exacerbated peripherally induced pain but produced nocifensive behavior indicative of peripheral nociceptive input even when no painful stimuli were applied. These experiments strongly suggest that there is a robust *endogenous* GABAergic inhibition in DRG. The exact mechanisms of GABAergic transmission in the afferent fibers as well as whether it is indeed restricted to their peripheral segments are yet to be elucidated.

**Figure 9:**
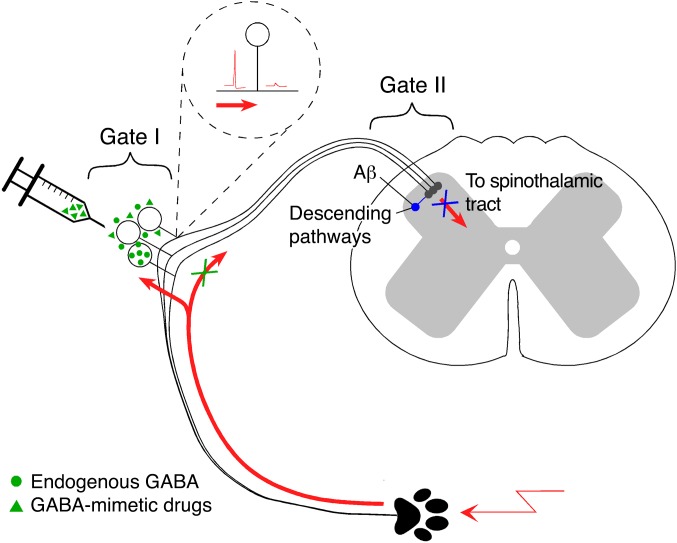
Schematic for peripheral somatosensory integration in DRG. Peripheral nociceptive stimulation triggers activity-dependent release of GABA from somatic/perisomatic compartments of afferent fibers located in DRG, which, in turn, depolarizes T-junctions of GABA_A_-expressing nociceptive fibers and lowers the AP safety factor at the T-junction. The latter effect results in the AP failure at the T-junction and reduced excitatory input into the spinal cord. A similar effect could be achieved by targeted delivery of GABA or GABA mimetics to the DRG or by the increasing of GABA tone with GABA reuptake inhibitor. Thus, DRG can be considered as a filter or an additional “gate” for nociceptive transmission.

While we cannot completely rule out a contribution of pre- or postsynaptic effects in the spinal cord to the antinociceptive effects of peripheral GABA, there are several arguments that such contribution is minimal. (a) Neither the fluorescent dye injected through the DRG cannula nor drugs that were injected in the same way (GABA, bicuculline, NO711) were detected in the spinal cord. (b) There were no contralateral effects of any DRG-injected drugs or optogenetic stimulation, suggesting that the application of drugs or light was indeed DRG-localized. (c) Application of glutamate to DRG produced unilateral antinociceptive effect, while intrathecal application produced bilateral pain. Thus, we believe that the majority of GABAergic communication reported here is somatic or perisomatic. Indeed, DRG soma is likely to be a most voluminous GABA reservoir, while the T-junction is the most likely site of the AP failure following GABA_A_ receptor activation. Our hypothesis is summarized in Figure 9: APs generated peripherally (at nerve endings or peripheral injury sites) invade the somatic/perisomatic compartment of sensory neurons (2, 3), which, in turn, triggers GABA release and causes autocrine or paracrine inhibition of the same or neighboring fibers due to the increased AP filtering at the corresponding T-junctions. The physiological outcomes of such inhibition can be the isolation of exact origin of pain (e.g., by suppressing the activity in fibers innervating bordering areas of the skin), suppression of subthreshold signals, and/or termination of nociceptive signal.

Abundance of receptors for other major neurotransmitters expressed at the somatic/perisomatic sites of sensory neurons and previously reported evidence of cross-excitation within the sensory ganglia (55, 56) may indicate that the DRG is indeed a sophisticated preliminary integrator of peripheral somatosensory information. Thus, our study proposes an additional mechanism to the broadly accepted gate control theory of pain, which postulated that integration of ascending peripheral and descending central pathways in the superficial dorsal horn can “gate” nociception (ref. 57 and Figure 9). Strong analgesic effects of focally applied GABA mimetics or GAT1 inhibitor (especially their efficacy in chronic pain models) suggest a possibility for therapeutic targeting of DRG for pain relief, a seemingly contentious idea that nevertheless is supported by earlier studies (14, 58). Indeed, recent and yet unexplained clinical evidence indicates that direct electrical stimulation (“neuromodulation”) of the DRGs in human neuropathic pain sufferers by the implanted stimulators provides efficacious pain relief even to the most resilient types of neuropathic pain (59–61). We hypothesize that analgesic efficacy of DRG neuromodulation is based on the engagement of the peripheral ganglionic “gate” (Figure 9); furthermore, we suggest that lasting pain relief can be achieved by peripherally acting GABA mimetics.

## Methods

### Neuronal cultures and slice preparation

DRG neurons from adult Sprague-Dawley rats (Beijing Vital River Laboratory Animal Technology Co. Ltd.) were dissociated as described previously (62). For sharp electrode recording, DRG slices were prepared from 12-day-old Wistar rats as described earlier (63). DRGs were embedded in agar and sliced (300 μm) in ice-cold extracellular solution using a vibrating blade microtome (VT100S, Leica Microsystems). Slices were then stored at room temperature for the remainder of the day in carbogenated (95% 0_2_/5% CO_2_) extracellular solution containing (in mM): 115 NaCl, 25 NaHCO_3_, 11 d-glucose, 5.6 KCl, 2 MgCl_2_, 1 NaH_2_PO_4_, 2.2 CaCl_2_ (pH 7.4).

### Acute focal application of drugs to DRG in vivo

All surgical procedures were performed under deep anesthesia with an i.p. injection of pentobarbital sodium (60–80 mg/kg) in accordance with the Animal Care and Ethical Committee of Hebei Medical University under the International Association for the Study of Pain guidelines. A DRG cannula for focal application of substances to DRG was implanted as previously described (28). Briefly, a midline incision was made at the L4–L6 spinal level of adult male rats (Sprague-Dawley; 180–200 g), and the L5 was identified at the midpoint of a link between both sides of the iliac crest. A 0.8-mm hole (~1 mm off the inferior edge of the transverse process) was drilled through the transverse process over the L5 DRG. Approaching of a ganglion was verified by the twitch of the paw. A hooked stainless steel blunt-tip cannula (inner diameter 0.64 mm, length 4 mm) was forced into the hole and connected to a polypropylene tube (inner diameter 0.41 mm, length 4.5 mm). The incision was closed with sutures, and the cannula was firmly fixed in place with dental cement. Intramuscular injection of benzylpenicillin (19 mg/0.1 ml) was given immediately after surgery. Procedure for mice was similar, but L4 DRG was used for implantation and 5 mg/25 μl of benzylpenicillin was used. Postoperatively, rats were housed individually in plastic cages with sawdust flooring and supplied with water and food ad libitum. Animals were left to recover for at least 24 hours before the experiments were carried out. Animals developing signs of distress were humanely sacrificed.

For continuous local delivery of drugs, osmotic minipumps (ALZET osmotic pump model 2002) were implanted in a procedure similar to the DRG cannula implant. The pumps were implanted s.c. in the neck while the cannula tube (ALZET Brain infusion kit 2) connected to the pump was inserted into the DRG hole. Before the implantation, the brain infusion assembly with attached osmotic pump was incubated in sterile saline (0.9%) at 37°C for 6 hours. This pump model releases approximately 0.5 μl/h for 14 days.

In order to verify that drug exposure was limited to the DRG, a fluorescent dye, CFSE (Sigma-Aldrich; 20 μM in 5 μl), was injected via the cannula. Approximately 30 minutes after injection, the animal was sacrificed, and the L5 DRG with the dorsal root and the associated lumbar spinal cord segment was excised, submerged in Tissue-Tek OCT (Sakura), frozen, and sectioned (15 μm) using a freezing microtome (CM1950, Leica). Slices were then analyzed for the presence of dye using confocal microscopy (TCS SP5 II, Leica).

### DREADD

VGAT-Cre (*Slc32a1^tm2(cre)Lowl^*) mice were from The Jackson Laboratory. AAV9-DIO-hM3Dq (Gq DREADD) viruses were obtained from Hanbio Biotechnology Co. Ltd. Virus injections into L4 DRG were performed under deep anesthesia with an i.p. injection of pentobarbital sodium (60–80 mg/kg). L4 DRGs were exposed by removal of both spinous and transverse processes of the vertebra bone. The microinjector (Hamilton Co.) was inserted into the ganglion to a depth of 500 μm from the exposed surface. The virus solution (5 μl) was injected slowly, and the needle was removed 2 minutes after the injection was complete. The muscles overlying the spinal cord were loosely sutured together, and the wound was closed. Animals were allowed to recover for at least 4 weeks before the experiments were carried out. Animals developing signs of distress were humanely sacrificed. Four to six weeks after injection of DREADD virus into the DRG of the VGAT-Cre or WT littermates, behavior tests were initiated, carried out 30 minutes after an i.p. injection of clozapine-*N*-oxide (CNO; dissolved in 0.9% saline, 5 mg/kg; Sigma-Aldrich) or saline. In some cases, after the behavior testing mice were humanely sacrificed and DRG extracted for mCherry visualization.

### In vivo optogenetic stimulation

VGAT-ChR2-EYFP mice were from The Jackson Laboratory. The implantation of optical fiber was performed similarly to the DRG cannula implant with modifications. A stainless steel cannula guide (RWD Life Science Co. Ltd., China; diameter 0.64 mm) was forced into the hole in the transverse process over the L5 DRG, and the optical fiber (RWD Life Science Co. Ltd., China; diameter 0.2 mm, length 1 m) was inserted through the guide. The incision was closed with sutures, and the fiber was firmly fixed in place with dental cement; the rest of the procedure was similar to the cannula implantation. Laser stimulation (473 nm, 3 mW, 30 Hz for 10 seconds with 20-second interval) was elicited using an MLL-FN-473-50 unit (Changchun New Industries Optoelectronics Technology Co., Ltd.) controlled by a pulsing set (S48 Stimulator, Grass Technologies, An Astro-Med, Inc. Product Group).

### Chronic pain models

Chronic constriction injury (CCI) was performed as described previously (64). Briefly, rats were anesthetized with an i.p. injection of sodium pentobarbital (60–80 mg/kg). The right hind leg was shaved and cleaned using 70% ethanol. The sciatic nerve was exposed by blunt dissection at the mid-thigh level, proximal to the sciatic trifurcation. Four nonabsorbable sterile surgical sutures (4–0 chromic gut) were loosely tied around the sciatic nerve with an approximately 1.0-to-1.5-mm interval between the knots. The skin was sutured, and the animal was transferred to a recovery cage. To induce chronic inflammatory pain, CFA (100 μl) was injected into the plantar surface of the right hind paw of the rat.

### Behavioral tests

In all tests, animals were habituated to the testing environment for 3 hours before behavioral testing. To evaluate the effect of focal application of GABA modulators to DRG on the nociceptive processing, compounds were injected via the DRG cannula in a volume of 5 μl immediately before the hind-paw injection of BK (200 μM) or CAP (20 μM). The volume of hind-paw injections in mice and rats was 20 μl and 50 μl, respectively. After the injections, animals were returned to the cage and video-recorded for 30 minutes. Time spent licking, biting, and flinching the injected paw was analyzed by the operator blind to the composition of the drug solution. Mechanical and thermal sensitivity were analyzed using the von Frey and Hargreaves methods, respectively (see Supplemental Methods).

### Electrophysiology

Perforated patch recordings in current- or voltage-clamp configurations were performed at room temperature. Currents were amplified and recorded using an EPC-10 patch amplifier and Patchmaster 2.2 software (HEKA Electronik) or an Axon Patch 700B amplifier and pClamp 10.0 software (Axon Instruments), and were sampled at 5 kHz. Liquid junction potentials were calculated using the pClamp and corrected (65). Continuous current-clamp recording with no current injection was used for monitoring of membrane potential (*V*_m_). Linear ramps of currents from 0 to 0.8 nA (1 second duration) were injected for measuring AP firing parameters. The extracellular solution contained (in mM): 160 NaCl, 2.5 KCl, 5 CaCl_2_, 1 MgCl_2_, 10 HEPES, and 8 glucose (pH 7.4). The intracellular solution contained (in mM): 150 KCl, 5 MgCl_2_, 10 HEPES (pH 7.4) (with 0.4 mg/ml gramicidin; Sigma-Aldrich).

Sharp electrode recordings were performed from DRG slices held in a submerged-type chamber and perfused with carbogenated extracellular solution (4–5 ml/min) at room temperature. Electrodes were pulled to resistances of 70–120 MΩ when filled with a recording solution (1 M K-acetate, 1 mM KCl; pH 7.2 adjusted with acetic acid). Recordings were made using a SEC-05L amplifier (npi electronic) and digitized (10 kHz) with a PC-based system (Digidata 1200 and Clampex 9.3, Molecular Devices) and analyzed off-line (Clampfit 10.1).

For sniffing patch recordings, HEK293 cells (ATCC) were transiently transfected with cDNA encoding human α_1_, β_2_, and γ_2_ subunits of GABA_A_ receptors (gift of David Weiss, Department of Physiology, University of Texas Health Science Center, San Antonio, Texas, USA) and GFP. The day before recording, transfected HEK293 cells were trypsinized and added to the coverslips containing 48-hour DRG cultures. Gramicidin-perforated patch recordings were made the next day from the coculture; transfected HEK293 cells were identified by GFP fluorescence.

For optical stimulation of DRG neurons from VGAT-ChR2-EYFP mice, a 473-nm blue light (3 mW) was elicited using the same device as for in vivo stimulation.

### Immunohistochemistry

GAD67-GFP mice (26) were transcardially perfused with 4% PFA under terminal anesthesia (sodium pentobarbital, 80 mg/kg). Lumbar DRGs were removed and stored in 0.1 M phosphate buffer (Sigma-Aldrich) followed by embedding in 10% porcine skin gelatin (Sigma-Aldrich), then postfixed in 4% PFA for 1 hour prior to sectioning. Thirty-five-micrometer DRG sections were cut using a vibrating microtome (Leica). Sections were washed once with 0.1 M PBS (Sigma-Aldrich) and blocked for 2 hours with blocking buffer (3% donkey serum in 0.1 M PBS; Sigma-Aldrich). Primary antibodies were diluted in 0.3% Triton X-100/PBS buffer before overnight incubation at 4°C. Detailed antibody information is given in Supplemental Table 5. The following day, sections received a further 3 washes in PBS before incubation with secondary antibodies and/or GFP booster (Chromotek) for 4 hours at room temperature. Sections were washed with PBS 3 times and placed on microscope slides in Vectashield with DAPI (Vector Laboratories). Staining was visualized using a confocal fluorescent microscope (LSM700, Zeiss). Tissue preparation of electron microscopy is described in Supplemental Methods.

### RT-PCR

Total RNA from all DRGs of 1 adult rat was extracted using a commercial RNA isolation kit (RNAiso, Takara). Isolated RNA was dissolved in 20 μl diethyl pyrocarbonate–treated (DEPC-treated) water and reverse-transcribed using an RT reagent kit (PrimeScript with gDNA Eraser, Takara) and a thermal cycler (Mastercycler, Eppendorf). Quantitative PCR reaction was performed using a kit (SYBR Premix Ex TaqII [Tli RNase H Plus], Takara), and the fluorescent DNA was detected and quantified with an FQD-48A(A4) system (BIOER). The PCR products were also run on a 2% agarose gel and were visualized using a gel imager (TFP-M/WL, Vilber Lourmat). A list of primers used in standard RT-PCR experiments is given in Supplemental Table 6.

For single-cell RT-PCR, neurons were aspirated into a patch pipette; the electrode tip was then broken into a PCR tube containing 0.7 μl of oligo-dT (50 mM), 1 μl of dNTP mixture (10 mM), 0.5 μl of MgCl_2_ (25 mM), 0.7 μl of RNaseOUT (40 U/μl; Invitrogen), and 1.4 μl of DEPC-treated water. The mixture was heated to 65°C for 5 minutes and then placed on ice for 1 minute. Single-strand cDNA was synthesized from the cellular mRNA by addition of 0.5 μl of RT buffer, 1.5 μl of MgCl_2_ (25 mM), 1 μl of DTT (1 M), 0.5 μl of RNaseOUT (40 U/μl), and 1 μl of SuperScript III RT (200 U/μl) and then incubation of the mixture at 55°C for 50 minutes followed by 85°C for 5 minutes. Synthesis of single-cell cDNA was performed using a C1000 Touch thermal cycler–CFX96 Real-Time PCR. First-strand synthesis was executed at 95°C (5 minutes) followed by 38 cycles (95°C for 1 minute, 60°C for 1 minute, 72°C for 1 minute) and a final 10-minute elongation at 72°C by addition of the specific “outer” primer pairs (Supplemental Table 7) into each PCR tube (final volume 60 μl). Then, 2.5 μl of the product of the first PCR was used in the second amplification round by use of specific “inner” primer (final volume 25 μl; Supplemental Table 7). The second amplification round consisted of heating the samples to 95°C for 5 minutes followed by 30 cycles (95°C for 1 minute, 60°C for 50 seconds, 72°C for 1 minute) and 10-minute elongation at 72°C. The products of the second PCR were analyzed in 2% agarose gels and stained with ethidium bromide. SuperScript III First-Strand Synthesis System Kit and GoTaq Green Master Mix were obtained from Invitrogen and Promega, respectively.

### GABA measurement with HPLC and LC–tandem mass spectrometry

#### GABA release from whole DRGs.

All DRGs from 1 adult rat were rapidly extracted into saline on ice and washed twice. Standard DRG incubation solution (150 μl) containing (in mM) 160 NaCl, 2.5 KCl, 5 CaCl_2_, 1 MgCl_2_, 10 HEPES, 8 d-glucose (pH 7.4) was added and incubated for 30 minutes in 37°C. GABA release was induced in the standard DRG incubation solution by the addition of ATP (1 mM), BK (100 μM), and CAP (1 μM), or by the equimolar substitution of 100 or 150 mM NaCl in the incubation solution with KCl for 30 minutes at 37°C. After incubation, 100 μl supernatant was removed and mixed with 300 μl acetonitrile and centrifuged (15,294 *g* for 10 minutes at 4°C). The supernatants were stored at –20°C until analysis.

#### GABA release from cultured DRG neurons.

Dissociated DRG cultures were plated onto glass coverslips coated with poly-d-lysine and laminin and cultured for 2–5 days. The culture medium was discarded and the cultures were washed with saline once; then 150 μl standard DRG incubation solution with or without added agonists, or 150 μl high-K^+^ solution, was added and incubated for 30 minutes at 37°C. The rest of the procedure was the same as for the whole-DRG tissue. Detailed description of HPLC and LC–tandem mass spectrometry conditions is provided in Supplemental Methods.

### Computer modeling

A computational model of an unmyelinated nociceptive neuron passing through the DRG was based on a model recently reported by us (28, 46). For detailed description see Supplemental Methods.

### Statistics

All data are given as mean ± SEM. Differences between groups were assessed by paired or unpaired 2-tailed Student’s *t* test. For data that failed normality testing, a paired-sample Wilcoxon signed rank test or Mann-Whitney test was used. Multiple groups were compared using 1-way ANOVA with Bonferroni correction or by Kruskal-Wallis ANOVA with Mann-Whitney test. Hyperalgesia data in chronic pain models were analyzed using 2-way ANOVA with Bonferroni correction. Differences were considered significant at *P* ≤ 0.05. Statistical analyses were performed using OriginPro 9.1 (Originlab Corp.). In bar charts shown in the figures, number of experiments is indicated within or above each bar; single, double, and triple asterisks indicate significant difference from the appropriate control with *P* ≤ 0.05, 0.01, or 0.001, respectively.

### Study approval

All animal experiments were approved by the Animal Care and Ethical Committee of Hebei Medical University (Shijiazhuang, China) and were in accordance with the International Association for the Study of Pain guidelines for animal use.

## Author contributions

XD designed experiments, performed electrophysiology experiments, and analyzed data. HH performed electrophysiology and in vivo experiments, and analyzed data. Y Yang, SH, and CW performed HPLC, immunohistochemistry, and PCR experiments, and analyzed data. SG performed DRG slice recordings and analyzed data. Y Yanagawa provided GAD67-GFP reporter mice and advised on corresponding experiments. JD, IE, and EJ performed immunostaining and electron microscopy, and analyzed data. RR performed VGAT immunostaining and analyzed data. XL performed single-cell PCR and analyzed data. JQ designed and supervised HPLC and HPLC–mass spectrometry measurements, and analyzed data. BG performed in vivo experiments and analyzed data. DBJ performed computer modeling. NG, XD, and HZ designed the study, supervised experiments, and analyzed data. NG wrote the manuscript, helped by all other authors, who also critiqued the manuscript for intellectual content.

## Supplementary Material

Supplemental data
