# Supplementary material for: Local GABAergic signaling within sensory ganglia controls peripheral nociceptive transmission
Source: J Clin Invest. 2017 Apr 4;127(5):1741–56. doi: 10.1172/JCI86812 (PMC5409786; doi:10.1172/JCI86812)
Supplement: Supplemental data [file jci-127-86812-s001.pdf]

Local GABAergic Signaling within Sensory Ganglia Controls Peripheral Nociceptive Transmission

Xiaona Du, Han Hao, Yuehui Yang, Sha Huang, Caixue Wang, Sylvain Gigout, Rosmaliza Ramli, Xinmeng Li, Ewa Jaworska, Ian Edwards, Jim Deuchars, Yuchio Yanagawa, Jinlong Qi, Bingcai Guan, David B. Jaffe, Hailin Zhang, Nikita Gamper

Supplementary materials

Supplementary Methods

Electron microscopy. Adult Wistar rats were transcardially perfused with 2% paraformaldehyde, 2% glutaraldehyde in 0.1M PB. L4 and L5 DRGs were embedded in 10% gelatin and sectioned at 100 µm on vibratome. Sections were permeabilised in 50% ethanol and incubated overnight with primary antibodies. Primary antibodies were detected with biotinylated secondary antibodies and ExtrAvidin peroxidase (Sigma), visualized with DAB kit (Vector SK-4100). Sections were then trimmed and further fixed in 50/50 3% potassium ferrocyanide and 4% osmium tetroxide for 1 hour. Sections were then incubated for 20 min in 1% thiocarbohydrazide bridging solution, then in a further 2% osmium tetroxide for 30 min. Sections were stained in uranyl acetate (4°C, overnight) then in lead aspartate (60°C, 30 min). Sections were then dehydrated using a series of ice cold ethanol followed by acetone and incubated in a mixture of Durcupan resin and acetone in increasing strength (finally in 100% resin overnight). Sections were then placed in fresh Durcupan resin for 2 hrs and mounted on a glass slides with aclar coverslips and placed in a polymerising oven at 60°C for 48 hours. Semi-thin 500 nm sections were cut on an ultramicrotome and stained with toluidine blue; 70 nm ultrathin sections were then cut and viewed on Tecnai-G2 Spirit TEM apparatus with LaB6 gun and equipped with Gatan 2k CCD camera, operating on 120kV.

HPLC and LC-MC/MC

Derivatization. For measurement of GABA using HPLC, chemical derivatization was performed. Briefly, 50µl sample or standards solutions was mixed with 50µl of o-phthalic aldehyde solution (OPA; pH 9.26). The derivatized samples were analyzed after 2min at room temperature.

HPLC equipment and conditions. Shimadzu HPLC system with an automatic sampler (SIL-20A), a column oven (CT-20A), a fluorescence detector (RF-10AXL), and a LC-20AT pump was used. The system was equipped with Intersil ODS3 chromatographic column (5 µm, 250mm×4.6 mm) and a pre-column. The mobile phase consisted of 0.1M sodium acetate (pH 4.62, adjusted with acetic acid) and methanol (55:45 for GABA or 73.5:26.5 for glutamate, v/v). Flow rate was 1.0 ml/min and column temperature was 35°C.

LC-MS/MS equipment and conditions. The LC-MS/MS analysis was performed on a Shimadzu Prominence LC-20A® HPLC systems and an AB Sciex 4000 QTRAP® hybrid triple quadrupole-linear Ion trap mass spectrometer equipped with an electrospray ionization source (ESI) operated in the positive ion mode. Instrument control and data acquisition were performed with AB Analyst 1.6.2 software. LC was performed on a Luna HILIC (3 µm, 100×4.6 mm) column at 35℃. The mobile phases were (A) 2 mM ammonium acetate, pH = 3 adjusted with formic acid, and (B) Acetonitrile with 2 mM ammonium acetate. Solvent ratio (A/B) of 25/75 (vol/vol) at a flow rate of 800 µl/min, and injection volumes of all the samples were 20µl. Quantitation was performed using the multiple reaction monitoring (MRM) mode to monitor protonated precursor to product ion transition of m/z 103.90 → 87.00 amu for GABA. The source dependent parameters maintained for analyte and IS were GS1: 50.0 psi, GS2: 50.0 psi, IS voltage: +4500.0 V, turbo heater temperature: 500.0°C, collision activation dissociation (CAD): high, curtain gas (CUR): 25.0 psi. The compound-dependent parameters such as declustering potential (DP) were optimized at 60.0 V, collision energy (CE) was 23.0 V, cell exit potential (CXP) was kept at 8.0 V and entrance potential (EP) was 10.0 V.

Behavioral tests. Mechanical withdrawal threshold was measured by a set of von Frey filaments (Stoelting Co, Chicago, IL, USA) with a calibrated range of bending force (1.4, 2, 4, 6, 8, 10, 15, and 26 g). Each rat was placed into a plastic cage with a wire mesh bottom. A single filament was applied

perpendicularly to the plantar surface of hind paw for five times with an interval of 5s. Positive response was defined as three clear withdrawal responses out of five applications. Filaments were applied in an up-and-down order according to a negative or positive response to determine the hind paw withdrawal threshold. Thermal withdrawal latency (Hargreaves method) was tested by a radiant heat lamp source (PL-200, Taimeng Co, Chengdu, China). The intensity of the radiant heat source was maintained at 25 ± 0.1%. Rats were placed individually into plexiglas cubicles placed on a transparent glass surface. The light beam from radiant heat lamp, located below the glass, was directed at the plantar surface of hindpaw. The time was recorded from the onset of radiant heat stimulation to withdrawal of the hindpaw. Three trials with an interval of 5 min were made for each rat, and scores from three trials were averaged.

Computer modeling. A computational model of an unmyelinated nociceptive neuron passing through the DRG was based on a model recently reported by us (1). The model was constructed and simulated using NEURON (http://www.neuron.yale.edu) (2, 3). Simulations were analyzed using IgorPro analysis software (Wavemetrics, Lake Oswego, OR). The soma was modeled as an isopotential cylinder with a 25μm diameter and length (4, 5), the stem axon had a diameter of 1.4μm and length of 75μm, while the diameters of the peripheral and central axons were 0.8 and 0.4 μm, respectively (6-9). Axonal compartments within the DRG were subdivided into 100 sections for computational accuracy (10). For all compartments E_{rest} = -60 mV, R_m = 10,000 Ωcm^2, C_m = 1 $\mu F/cm^2$, and R_a = 100 Ωcm(8). Voltage-gated Na^+ and K^+ channels were expressed in all compartments with a density of 0.04 S/cm^2, except for the soma where Na^+ channel conductance was 0.02 S/cm^2.

Voltage-gated Na^+ Channels: Voltage-dependence was adjusted to be approximately mid-way between values reported for Na_V1.7 and Na_V1.8 channels in DRG (11, 12). Current was calculated as $I_{Na} = \bar{g}_{Na} \cdot m^3 h \cdot (V - E_{Na})$, where $\bar{g}_{Na}$ is the Na^+ conductance density, E_{Na} = 50 mV and channel kinetics were described by the following equations: $\alpha_m = 0.554 \cdot (-44.9-V)/(\exp[(-44.9-V)/4]-1)$, $\beta_m = 0.485 \cdot (v-18)/\exp[(v-18)/5]-1)$, $\alpha_h = 0.221 \cdot (\exp[(-55-V)/18])$, $\beta_h = 6.9282 \cdot /(\exp[(-32-V)/5]+1)$, where $m_\infty = \alpha_m/(\alpha_m + \beta_m)$, $\tau_m = 1/(\alpha_m + \beta_m)$ and $h_\infty = \alpha_h/(\alpha_h + \beta_h)$, $\tau_h = 1/(\alpha_h + \beta_h)$, and $dm/dt = (m_\infty - m)/\tau_m$ and $dh/dt = (h_\infty - h)/\tau_h$.

Voltage-gated K Channels: The conductance was based on the generic Borg-Graham K_V model(13). Current was calculated as $I_K = \bar{g}_K \cdot n^3 l \cdot (V - E_K)$, where $\bar{g}_K$ is the conductance density, E_K = -90 mV and channel kinetics were described by the following equations: $\alpha_n = \exp[-0.1883 \times 10^{-5} \cdot (V+32)]$, $\beta_n = \exp[-0.0753 \cdot (V+32)]$, $\alpha_l = \exp[0.0753 \cdot (V+61)]$, $\beta_l = \exp[0.0753 \cdot (V+61)]$, $n_\infty = 1/(1+\alpha_n)$, $\tau_n = \beta_n/(0.052 \cdot (1+\alpha_n))$ and $l_\infty = 1/(1+\alpha_l)$, $\tau_l = \beta_n/(0.0017 \cdot (1+\alpha_l))$, where $dn/dt = (n_\infty - n)/\tau_n$ and $dl/dt = (l_\infty - l)/\tau_l$.

Supplementary References

1. Du, X., Hao, H., Gigout, S., Huang, D., Yang, Y., Li, L., Wang, C., Sundt, D., Jaffe, D.B., Zhang, H., et al. 2014. Control of somatic membrane potential in nociceptive neurons and its implications for peripheral nociceptive transmission. *Pain* 155:2306-2322.
2. Hines, M.L., and Carnevale, N.T. 1997. The NEURON simulation environment. *Neural Comput* 9:1179-1209.
3. Hines, M.L., and Carnevale, N.T. 2000. Expanding NEURON's repertoire of mechanisms with NMODL. *Neural Comput* 12:995-1007.
4. Hayar, A., Gu, C., and Al-Chaer, E.D. 2008. An improved method for patch clamp recording and calcium imaging of neurons in the intact dorsal root ganglion in rats. *J Neurosci Methods* 173:74-82.
5. Zhang, J.M., Donnelly, D.F., and LaMotte, R.H. 1998. Patch clamp recording from the intact dorsal root ganglion. *J Neurosci Methods* 79:97-103.
6. Ha, H. 1970. Axonal bifurcation in the dorsal root ganglion of the cat: a light and electron microscopic study. *J Comp Neurol* 140:227-240.
7. Hoheisel, U., and Mense, S. 1987. Observations on the morphology of axons and somata of slowly conducting dorsal root ganglion cells in the cat. *Brain Res* 423:269-278.
8. Luscher, C., Streit, J., Quadroni, R., and Luscher, H.R. 1994. Action potential propagation through embryonic dorsal root ganglion cells in culture. I. Influence of the cell morphology on propagation properties. *J Neurophysiol* 72:622-633.
9. Suh, Y.S., Chung, K., and Coggeshall, R.E. 1984. A study of axonal diameters and areas in lumbosacral roots and nerves in the rat. *J Comp Neurol* 222:473-481.
10. Segev, I., Fleshman, J.W., Miller, J.P., and Bunow, B. 1985. Modeling the electrical behavior of anatomically complex neurons using a network analysis program: passive membrane. *Biol Cybern* 53:27-40.
11. Cummins, T.R., Sheets, P.L., and Waxman, S.G. 2007. The roles of sodium channels in nociception: Implications for mechanisms of pain. *Pain* 131:243-257.
12. Sheets, P.L., Jackson, J.O., 2nd, Waxman, S.G., Dib-Hajj, S.D., and Cummins, T.R. 2007. A Nav1.7 channel mutation associated with hereditary erythromelalgia contributes to neuronal hyperexcitability and displays reduced lidocaine sensitivity. *J Physiol* 581:1019-1031.
13. Borg-Graham, L.J. 1987. Modeling the somatic electrical behavior of hippocampal pyramidal neurons. In *Department of Electrical Engineering and Computer Science*: Massachusetts Institute of Technology.

Supplementary Table 1. Expression of GAD65, GAD67, VGAT, GAT1 and TRPV1 in DRG neurons as detected by single-cell RT-PCR

	GAD65	GAD67	VGAT	GAT1	TRPV1
**GAD65**	10 (118)	4	5	6	5
**GAD67**	4	10 (118)	1	7	4
**VGAT**	5	1	9 (118)	4	1
**GAT1**	6	7	4	48 (99)	27
**TRPV1**	5	4	1	27	30 (99)

Number in each cell represents total number of neurons in which proteins listed in the top row and left column are co-identified. Grey cells show total number of neurons expressing the corresponding protein; the number of total neurons tested is given in parentheses.

Supplementary Table 2. Somatic diameter distribution of VGAT-positive DRG neuron cell bodies

Cell body size	Total number of neurons	% of total neurons	Number of VGAT-positive neurons	% of VGAT-positive neurons within the size band
<27 μm	197	38	29	15
27-30 μm	70	14	11	16
31-40 μm	129	25	26	20
41-50 μm	93	18	28	30**
>51 μm	26	5	8	31*
Total number of neurons	515		102	

*,** indicate significant difference from the proportion of VGAT-positive small-diameter (<27 μm) neurons at $p<0.05$ and $p<0.01$, respectively; Fisher's exact test. Data were obtained from three independent preparations, each from a separate rat.

Supplementary Table 3. Effect of GABA on small DRG neuron excitability

Parameter / Type of the response	Vm (mV)					Threshold current (pA)			Threshold voltage (mV)		
	Control	Peak	Steady-state	ΔVm (peak)	ΔVm (steady -state)	Control	Peak	Steady-state	Control	Peak	Steady-state
Firing abolished (GABA; n=11)	-59.8±1.8	-49.7±2.8 p≤0.001	-56.7±2.6 p=0.09	10.0±1.8	3.0±1.6	140.8±53.1	>800 p≤0.001	180.7±75.6 p≤0.01	-33.4±3.7	-	-29.1±4.4
Firing reduced (GABA; n=17)	-61.6±1.7	-49.9±2.0 p≤0.001	-58.4±1.8 p≤0.01	11.7±1.3	3.8±1.0	128.9±26.9	197.5±44.2 p=0.08	141.2±32.8	-23.3±4.1	-12.0±4.9 p≤0.05	-22.4±4.1
Firing increased (GABA; n=5)	-61.4±4.8	-41.3±1.9 p≤0.05	-53.2±4.8 p≤0.05	20.0±5.2	8.1±2.7	290.2±73.3	196.2±28.5	200.3±63.8	-3.0±7.3	-9.0±4.6	-0.2±4.2
All neurons (GABA; n=38)	-60.8±1.2	-48.9±1.4 p≤0.001	-57.3±1.4 p≤0.001	11.8±1.3	5.0±1.5	N/A[£]	N/A[£]	N/A[£]	N/A[£]	N/A[£]	N/A[£]
All neurons (muscimol; n=16)	-58.2±2.2	-49.1±2.1 p≤0.001	-54.9±2.5 p≤0.01	9.0±1.4	3.3±0.9	N/A[£]	N/A[£]	N/A[£]	N/A[£]	N/A[£]	N/A[£]

p values denote significant difference from control with paired two-tailed t-test; where no p value is given there was no statistical difference.

[£] Not analyzed in the total population of neurons tested due to significant number of neurons in which firing was abolished.

Supplementary Table 4. Effect of GABA on small DRG neuron excitability (continued)

Parameter / Type of the response	AP number			AP duration (ms)			AP amplitude (mV)			AHP amplitude (mV)		
	Control	GABA (peak)	GABA (steady-state)	Control	GABA (peak)	GABA (steady-state)	Control	GABA (peak)	GABA (steady-state)	Control	Peak	Steady-state
Firing abolished (GABA; n=11)	7.3±1.4	0 p≤0.001	4.4±1.3 p≤0.01	15.3±2.7	---	15.3±2.7 p≤0.05	43.7±3.9	---	37.6±4.3 p≤0.01	24.7±4.0	---	15.3±2.3 p≤0.001
Firing reduced (GABA; n=17)	14.8±2.2 $	9.5±2.0 p≤0.001	11.4±1.7 p≤0.01	16.5±1.2	18.0±2.0	17.1±1.5	46.8±3.3	33.9±7.7 p≤0.01	42.4±3.6 p≤0.01	31.3±2.0	25.5±1.9 p≤0.01	27.4±1.8 p≤0.01
Firing increased (GABA; n=5)	12.8±4.5	15.6±6.2	13.0±3.6	16.3±3.2	17.4±4.1	19.6±3.9 p≤0.05	44.3±7.5	42.5±8.2	36.3±6.3 p=0.06	35.8±2.1	29.6±4.7 p=0.09	32.3±3.9
All neurons (GABA; n=38)	11.9±1.5	7.6±1.7 p≤0.001	9.5±1.3 p≤0.001	N/A£	N/A£	N/A£	N/A£	N/A£	N/A£	N/A£	N/A£	N/A£
All neurons (muscimol; n=16)	9.7±2.4	6.6±2.2 p≤0.001	7.7±2.4 p≤0.001	N/A£	N/A£	N/A£	N/A£	N/A£	N/A£	N/A£	N/A£	N/A£

p values denote significant difference from control with paired two-tailed t-test; where no p value is given there was no statistical difference.

$ Different from the mean AP number (control conditions) in neurons where GABA application abolished firing at p≤0.05 (un-paired, two-tailed t-test)

£ Not analyzed in the total population of neurons tested due to significant number of neurons in which firing was abolished.

Supplementary Table 5. List of antibodies used in immunohistochemical experiments

Antibody	Produced in	Source/cat. #	Dilution
Anti-NF200	mouse	Sigma/N2912	1:2000
Anti-SV2	mouse	DSHB/AB2315387	1:1000
Anti-TRPV1	guinea pig	Neuromics/GP14100	1:100
Anti-TRPV1	rabbit	Abcam/ab10296	1:1000
Anti-VGAT	rabbit	Synaptic Systems/131002	1:1000 1:2000
Anti-VGAT	guinea pig	Synaptic systems/131/004	1:1000
Anti-GABA	rabbit	Sigma/A2052	1:10000
Anti-S100B	rabbit	Abcam/EP1576Y	1:1000
IB4	Griffonia Simplicifolia	Invitrogen/I21411	1:50
Anti-c-Fos	rabbit	Santa Cruz/gc-52	1:400
GFP booster	-	Chromotek/ATTO 488	1:200
Anti-TrkC	Goat	R&D Systems/ AF1404	1:500
Anti-Cre	rabbit	Abcam/ab190177	1:200
Secondary anti-mouse Alexa Fluor 555	Donkey	Life Technologies/ A31570	1:1000
Secondary anti-rabbit Alexa Fluor 555	Donkey	Life Technologies/ A31572	1:1000
Secondary anti-guinea pig Alexa Fluor 555	Goat	Life Technologies/ A21435	1:1000
Secondary anti-guinea pig Alexa Fluor 488	Goat	Life Technologies/ A11073	1:1000

Supplementary Table 6. List of primers used in standard RT-PCR experiments

Protein	Primers	Length (bp)
α1	F 5’-TGTCTTTGGAGTGACGACCG-3’<br>R 5’-ATCCCACGCATACCCTCTCT-3’	187
α2	F 5’-AGGTTGCTCCTGATGGCTCTA-3’<br>R 5’-TCTCGCTGGCACCGATTCT-3’	227
α3	F 5’-CTCCCAGTGCTTCTTCAACTCC-3’<br>R 5’-CTGCCACTATTATCTACTGTTTGCG-3’	231
α5	F 5’-CAAAACGCTCCTTGTCTTCTG-3’<br>R 5’-TGTGATGTTGTCATTGGTCTCA-3’	106
β1	F 5’-TGTGTTCGTGTTCCTGGCTCTACT-3’<br>R 5’-GCATCAACCTGGACTTTGTTCATC-3’	147
β2	F 5’-TGACCACAATCAATACCCATCT-3’<br>R 5’-ACAAAGACAAAGCACCCCATTA-3’	94
β3	F 5’-CTGTACGGGCTCAGGATCAC-3’<br>R 5’-GGGAGCTCGATCCTTTCCAC-3’	179
γ1	F 5’-AGACGGATGGGCTATTTCACAA-3’<br>R 5’-ATACCCAGGGATGTTCTAGCAGG-3’	119
γ2	F 5’-CGCAGTTCTGTTGAAGTGGG-3’<br>R 5’-CAGGGAATGTAGGTCTGGATGG-3’	179
γ3	F 5’-TGGTCTATTGGGTTGGATACCT-3’<br>R 5’-CACTACTTGTCTGGGGATGATG-3’	104
GAT1	F 5’-GATGGACTGGAAAGGTGGTCTA-3’<br>R 5’-CATTGTTGTGGAAAGAGTTGTAGC-3’	246
GAT2	F 5’-AGCGCTGGTGGACATGTATC-3’<br>R 5’-TGTGAGCATAATGAGCCCGA-3’	109
GAT3	F 5’-CTACCCCAAGGCTGTCACTATG-3’<br>R 5’-GGCTCTCCACACACACAAACT-3’	110
GAD65	F 5’-AAAGGTGGCGCCAGTGATTA-3’<br>R 5’-ACAAATCTTGTCCCAGGCGT-3’	176
GAD67	F 5’-CTTGTGAGTGCCTTCAGGGAG-3’<br>R 5’-CTTGCGGACATAGTTGAGGAGTA-3’	204

B1	F 5’-TCATCGGGTGGTATGCTGAC-3’<br>R 5’-GTTGGAAATGCTTCGGGTGT-3’	146
B2	F 5’-CTGGGCAAAATCATCCTCAAT-3’<br>R 5’-GACCTTCACCTCTCTGCTGTCT-3’	135
GAPDH	F 5’-GACATGCCGCCTGGAGAAAC-3’<br>R 5’-AGCCCAGGATGCCCTTTAGT-3’	
VGAT	F 5’-ACGACAAACCCAAGATCACG-3’<br>R 5’-AAGATGATGAGGAACAACCCC-3’	130

Supplementary Table 7. List of primers used in single-cell RT-PCR experiments

Protein	primers	Length (bp)
GAPDH	Fo 5'-CTAGAGACAGCCGCATCTTCTT-3'<br>Ro 5'-CCCTTTAGTGGGCCCTCGG-3'	861
	Fi 5'-CCAGCCTCGTCTCATAGACA-3'<br>Ri 5'-CGCTCCTGGAAGATGGTGAT-3'	255
GAD65	Fo 5'-TTGGGGTGGAGGGTTACTGA-3'<br>Ro 5'-AACTGGACTCTACTGTGACACC-3'	790
	Fi 5'-AAAGGTGGCGCCAGTGATTA-3'<br>Ri 5'-ACAAATCTTGTCCCAGGCGT-3'	167
GAD67	Fo 5'-GGCCTGAAGATCTGTGGTTT-3'<br>Ro 5'-TATGGCTCCCCCAGGAGAAA-3'	633
	Fi 5'-CTTGTGAGCGCCTTCAGGGAG-3'<br>Ri 5'-CTTGCGGACATAGTTGAGGAGTA-3'	204
VGAT	Fo 5'-ATTCAGGGCATGTTCGTGCT-3'<br>Ro 5'-ATGTGTGTCCAGTTCATCAT-3'	650
	Fi 5'-ATTCAGGGCATGTTCGTGCT-3'<br>Ri 5'-TGATCTGGGCCACATTGACC-3'	250
Gat-1	Fo 5'-AGTGTGACAACCCCTGGAAC-3'<br>Ro 5'-AGGTAGGACACGATGCACAC-3'	811
	Fi 5'-GCGCAACATGCACCAAATGACA-3'<br>Ri 5'-AGACCACCTTTCCAGTCCATCCAA-3'	140
Trpv1	Fo 5'-TGGAGTCCACACCACACAAG-3'<br>Ro 5'-GGTTCCCTAAGCAGACCACC-3'	746
	Fi 5'-GAGCAAGAACATCTGGAAG-3'<br>Ri 5'-GTGTTCCAGGTAGTCCAGTT-3'	186

Fo, Ro – outer primer pair; Fi, Ri – inner primer pair.

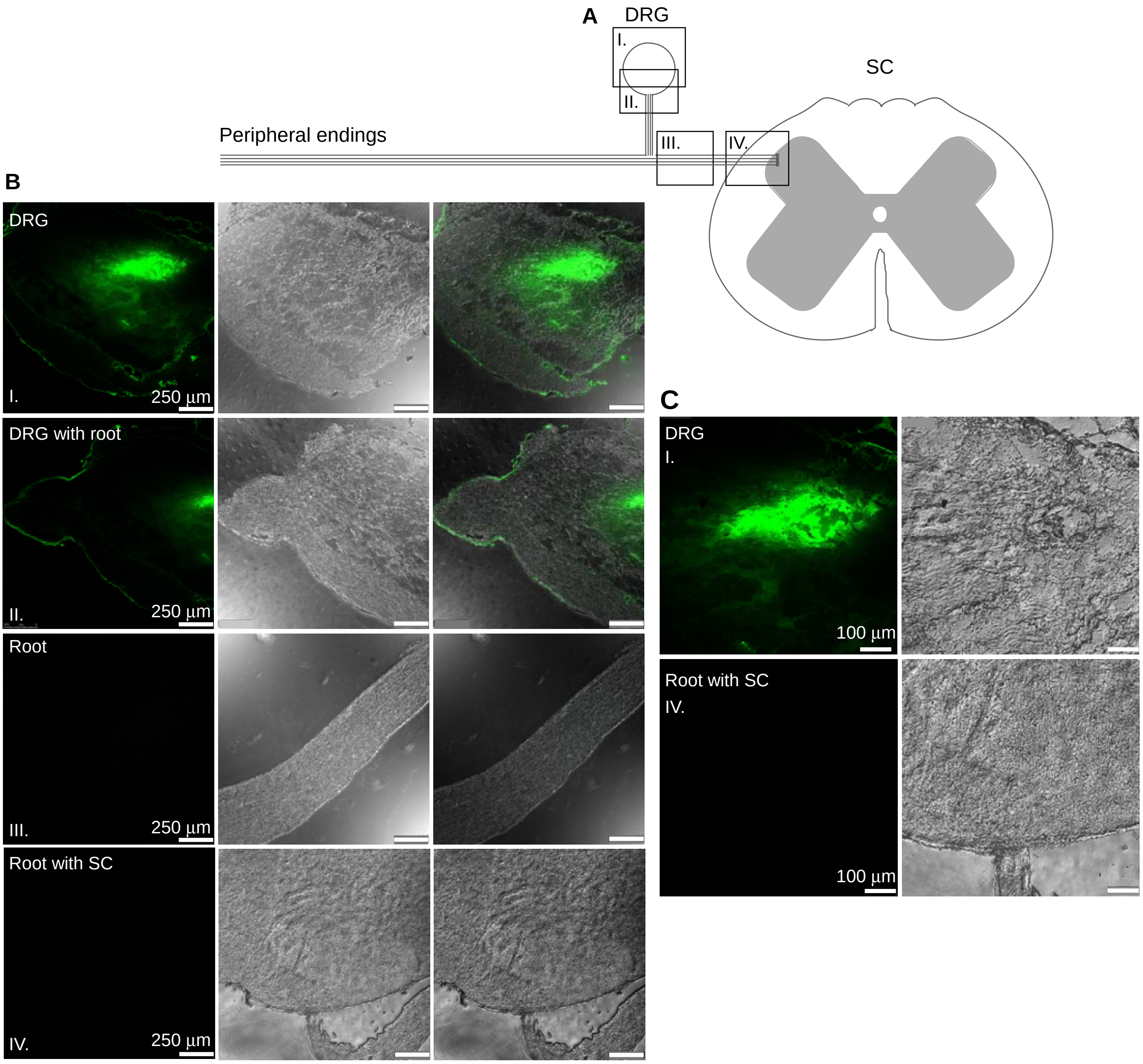

Supplementary Figure 1. Testing the DRG cannula injection accuracy. An extended dataset for the experiment shown in Fig 4A. **A** Schematic representation of a DRG neuron, rectangle frames I-IV depict location of tissue sections shown in **B**. **A** fluorescent dye, 5(6)-Carboxyfluorescein diacetate N-succinimidyl ester (20 µM in 5 µl), was injected via the implanted DRG cannula; 30 min after injection the animal was sacrificed, the L5 DRG with the dorsal root and the associated lumbar spinal cord segment were excised and analyzed for the presence of the dye using fluorescence microscopy. Higher-magnification images (as indicted by the scale bars) of segments I and IV are shown in **C**. Sections of segments I and IV are the same as those shown in Fig. 4A (bottom). Micrographs within each row are of the same magnification; scale bars are labeled on the left image in each row.

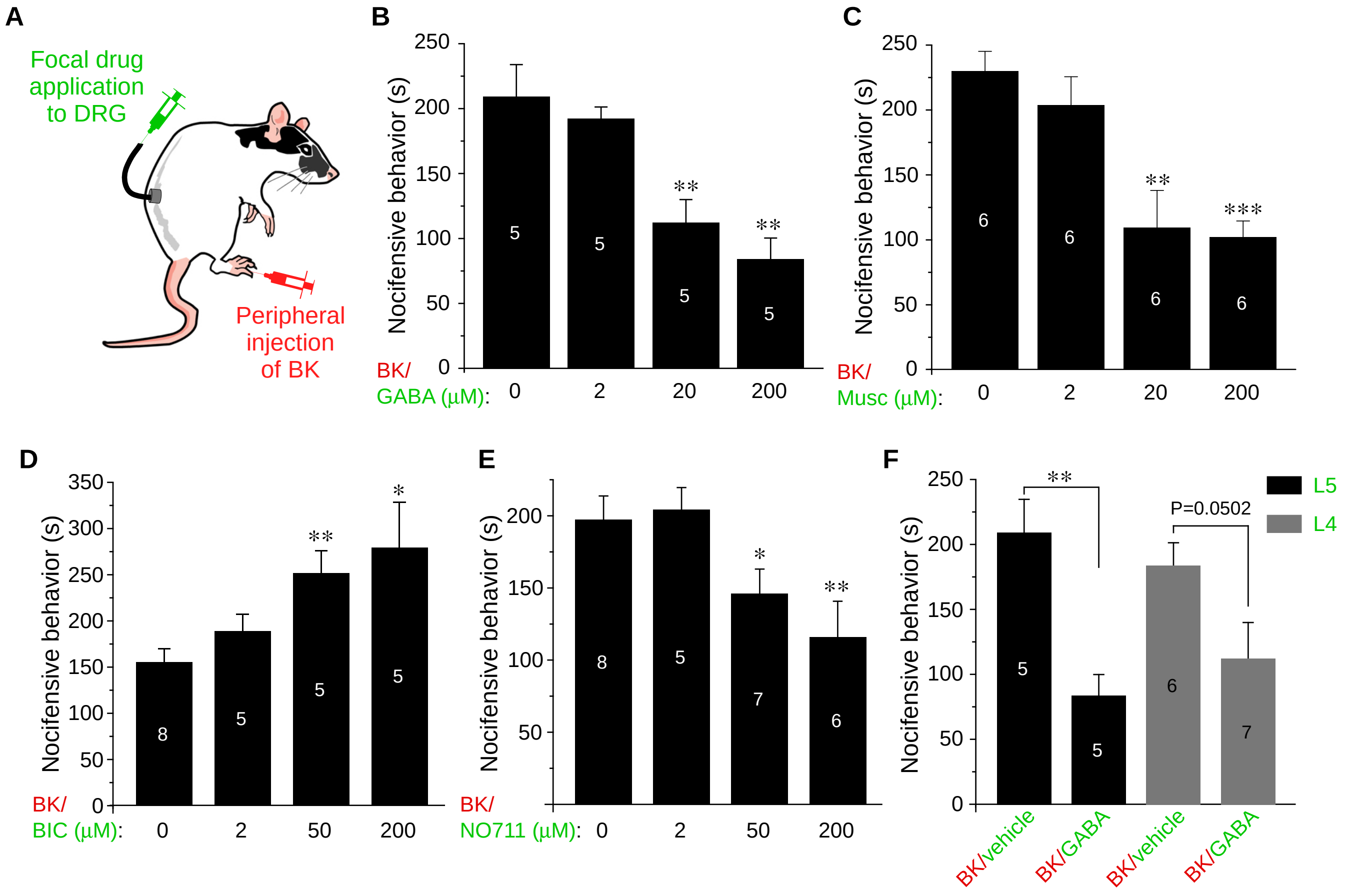

Supplementary Figure 2. Concentration dependence of the *in vivo* effects of the DRG-applied GABA modulators and the effect of GABA applied to the L4 DG. a Schematic of the drug administration. **B-E** Concentration dependency of the behavioral effects of the following compounds focally applied via the DRG cannula: GABA (**B**), muscimol (**C**), bicuculline (**D**) and NO711 (**E**). **F** Focal DRG application of GABA (200 µM, 5 µl) via L5 (black bars) and L4 (gray bars) DRG cannulas reduced pain produced by hind-paw injection of bradykinin (BK, 200 µM, 50 µl). *,**,*** indicate significant difference from the appropriate control with $p > 0.05$, 0.01 or 0.001 (one-way ANOVA with Bonferroni correction was used for multiple groups comparison and Mann-Whitney test for two-group comparison in **B**).

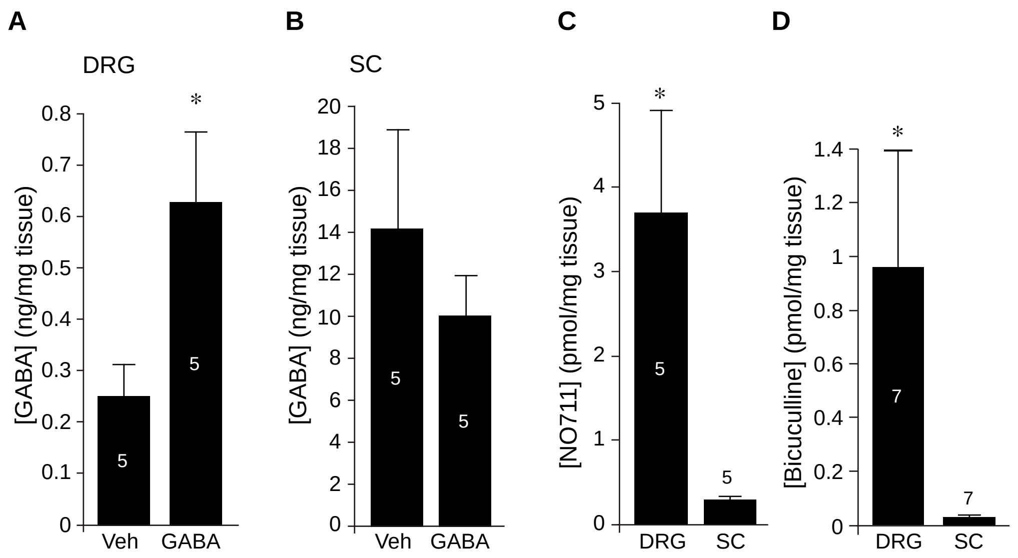

Supplementary Figure 3. HPLC analysis of focal DRG injections. **A**, **B** Analysis of GABA content in DRG (**A**) and spinal cord (**B**) after the focal injection of vehicle control or GABA via the DRG cannula. **C** HPLC-MS analysis of NO711 in DRG and spinal cord 15 min after the focal injection of the drug via the DRG cannula. **D** HPLC-MS analysis of bicuculline in DRG and spinal cord 15 min after the focal injection of the drug via the DRG cannula. In bar charts number of experiments is indicated within or above each bar; *, indicates significant difference from the appropriate control with $p > 0.05$ (unpaired, two-tailed t-test).

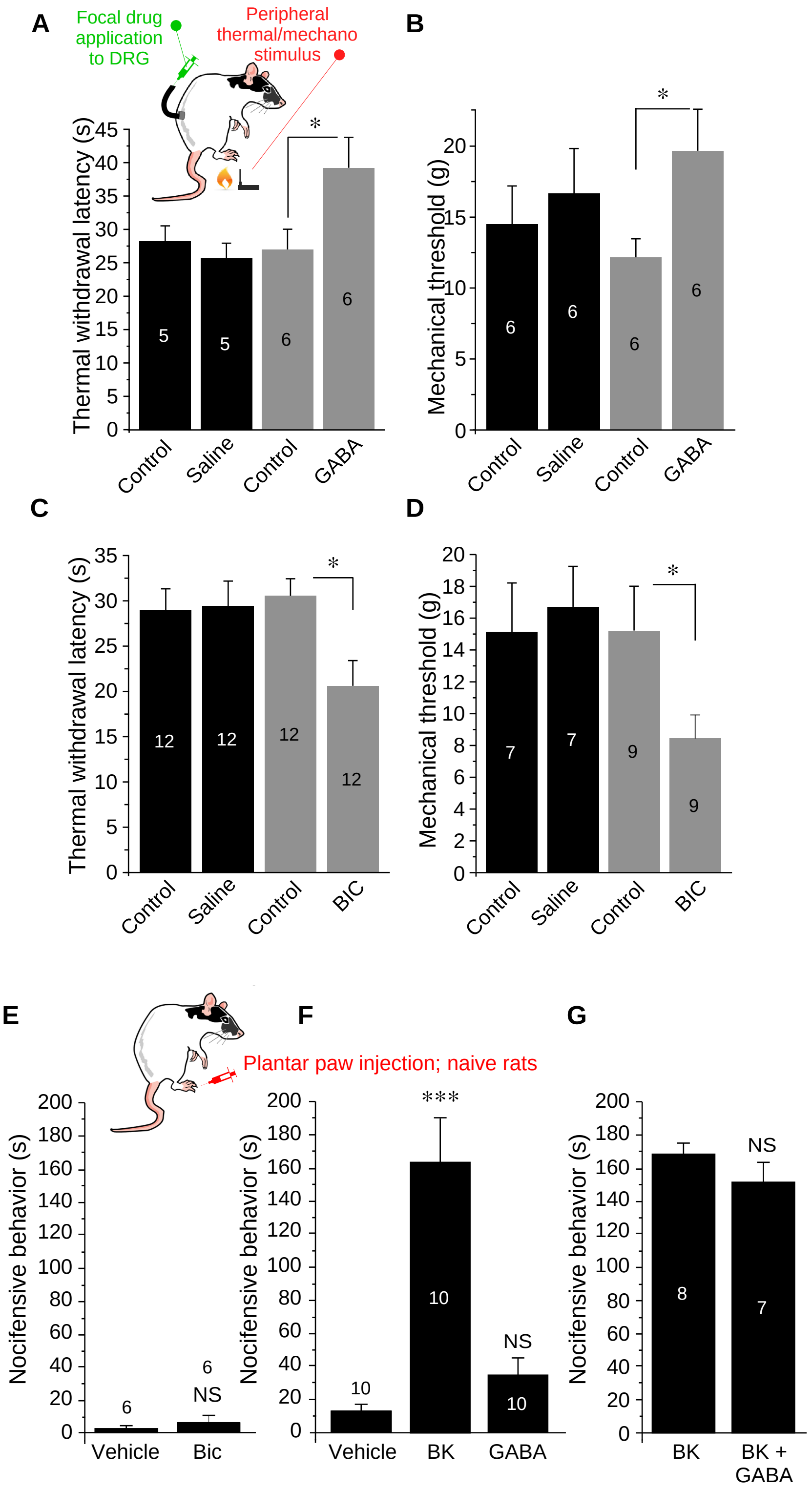

Supplementary Figure 4. Additional test of the peripheral effects of GABA. A-D Focal application of GABA to DRG affects sensitivity to noxious stimuli. Focal DRG application of GABA (200 µM, 5 µl; **A**, **B**) increased while application of $GABA_A$ receptor antagonist bicuculline (BIC, 200 µM, 5 µl, **C**, **D**) reduced hind paw sensitivity to thermal and mechanical stimulation. (**A**, **C**) show mean withdrawal latencies upon presentation with radial heat stimulus; (**B**, **D**) show mean withdrawal thresholds upon presentation with von Frey hairs. *, indicate significant difference from the appropriate control at p<0.05 (Mann-Whitney test). **E-G** Plantar paw injections of GABA modulators do not significantly affect acute nociception. **B** Peripheral plantar injections of bicuculline did not produce nocifencive beghaviour. (**F**, **G**) Peripheral plantar injections of GABA produced no nocifensive behavior (**F**) and did not affect BK-induced pain (**G**). ***, indicates significant difference from the appropriate control at p<0.001 (one-way ANOVA with Bonferroni correction). Number of experiments is indicated within or above each bar.

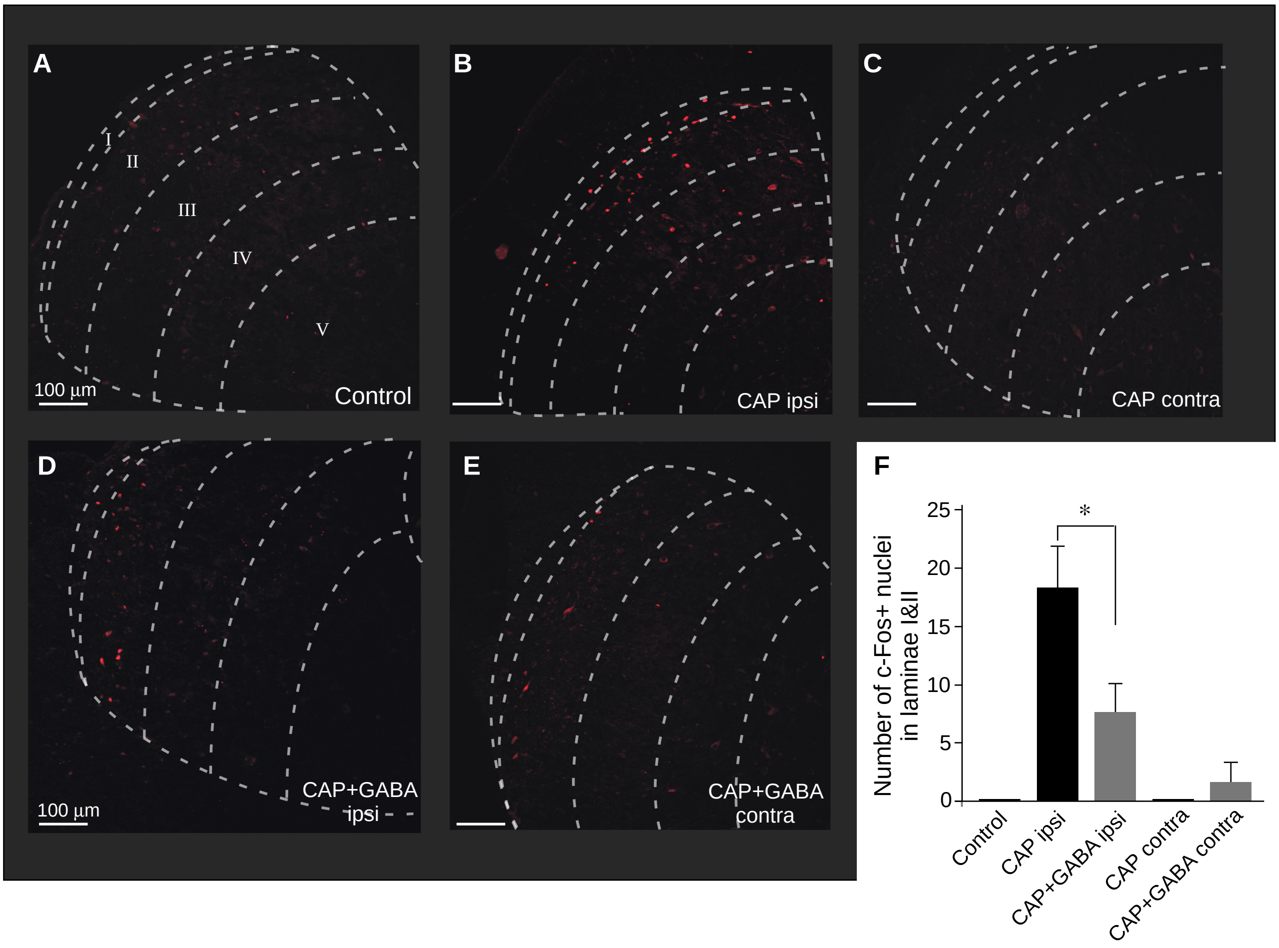

Supplementary Figure 5. Focal application of GABA to DRG reduces capsaicin-induced c-Fos expression in superficial spinal cord. A-E Exemplary c-Fos immunolabelling of the spinal cord sections form the following animals: (**A**) a naive rat; (**B**, **C**) a rat that received capsaicin injection (20 µM, 50 µl) to the hind paw (ipsi- and contralateral spinal cord sections are shown in **B** and **C** respectively); (**D**, **E**) a rat that received capsaicin (20 µM, 50 µl) injection to the hind paw and GABA (200 µM, 5 µl) into the DRG cannula (ipsi- and contralateral spinal cord sections are shown in **D** and **E** respectively). Laminae are indicated with white dashed lines and labelled in **A**. All micrographs are of the same magnification; scale bars are labeled on the left image in each row. **F** Summary of the experiment shown in **A-E**. *, indicates significant difference from the appropriate control at $p<0.05$ (unpaired, two-tailed t-test, n=3).

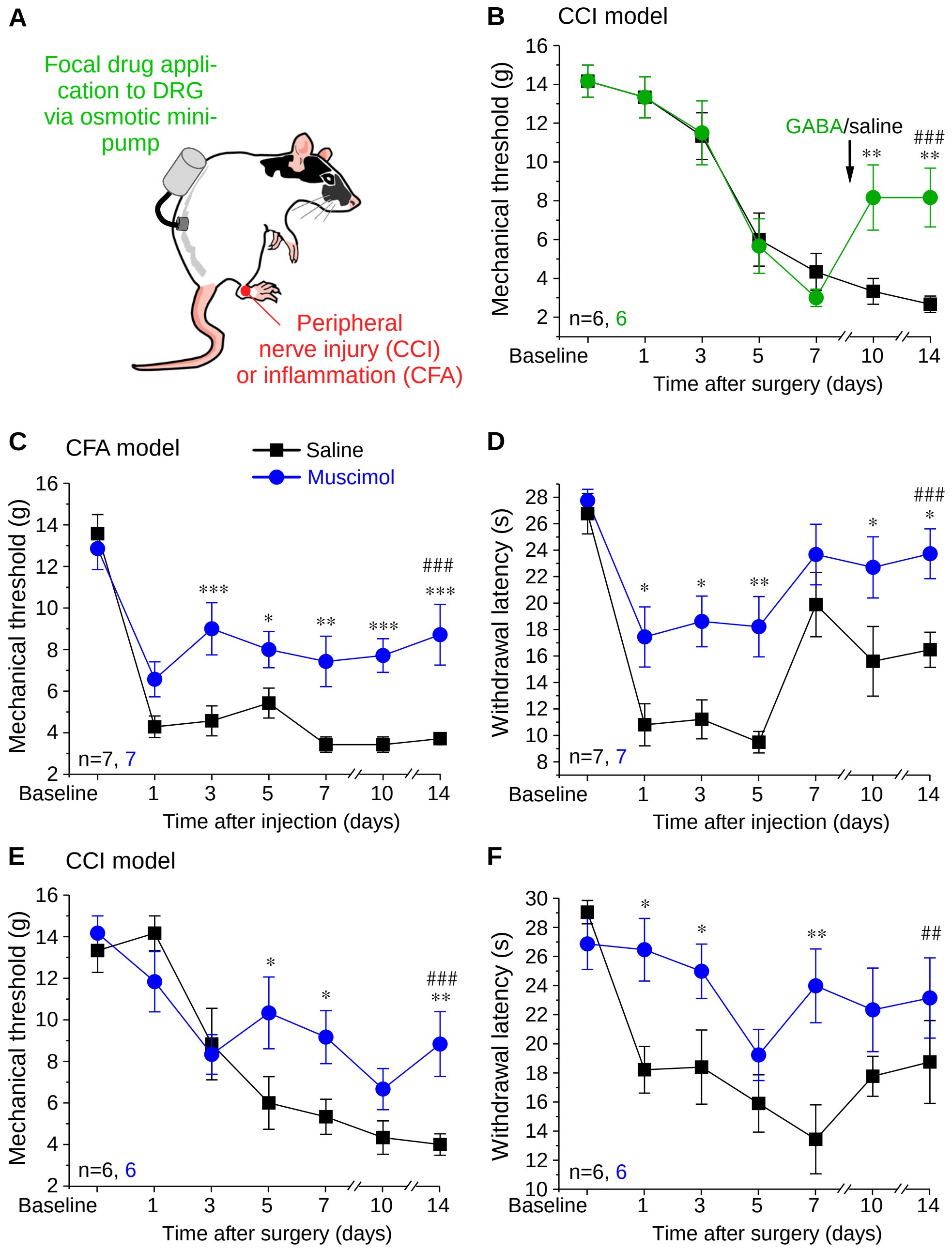

Supplementary Figure 6. Focal DRG infusion of GABA-mimetics alleviates chronic pain *in vivo*. A Schematic of the experiments on rats with DRG mini-pump implant. **B** Focal DRG application of GABA (200μM, ~0.5μl/hr) via osmotic mini-pump significantly alleviated mechanical hyperalgesia produced by chronic constriction injury (CCI). The pumps were implanted on day 8 after the injury. **C-F** Infusion of muscimol via the DRG mini-pump (200μM, ~0.5μl/hr) significantly reduced chronic mechanical (**C**, **E**) and thermal (**D**, **F**) hyperalgesia induced by plantar injection of complete Freund's adjuvant (CFA; 100 μl, **C, D**) or CCI (**E, F**). In **C**, **D** CFA/CCI porcedure and the mini-pump implantation were performed at the same time. #, ##, ### indicate significant difference at p<0.05, p<0.01, p<0.001 between saline and drug datasets with *, **, *** indicating the difference between groups within the corresponding time point (two-way ANOVA with Bonferroni correction for saline *vs.* drug comparison or one-way ANOVA with Fisher LSD post-hoc test for comparison between the groups). Number of experiments in B-F is indicated as n in each panel.

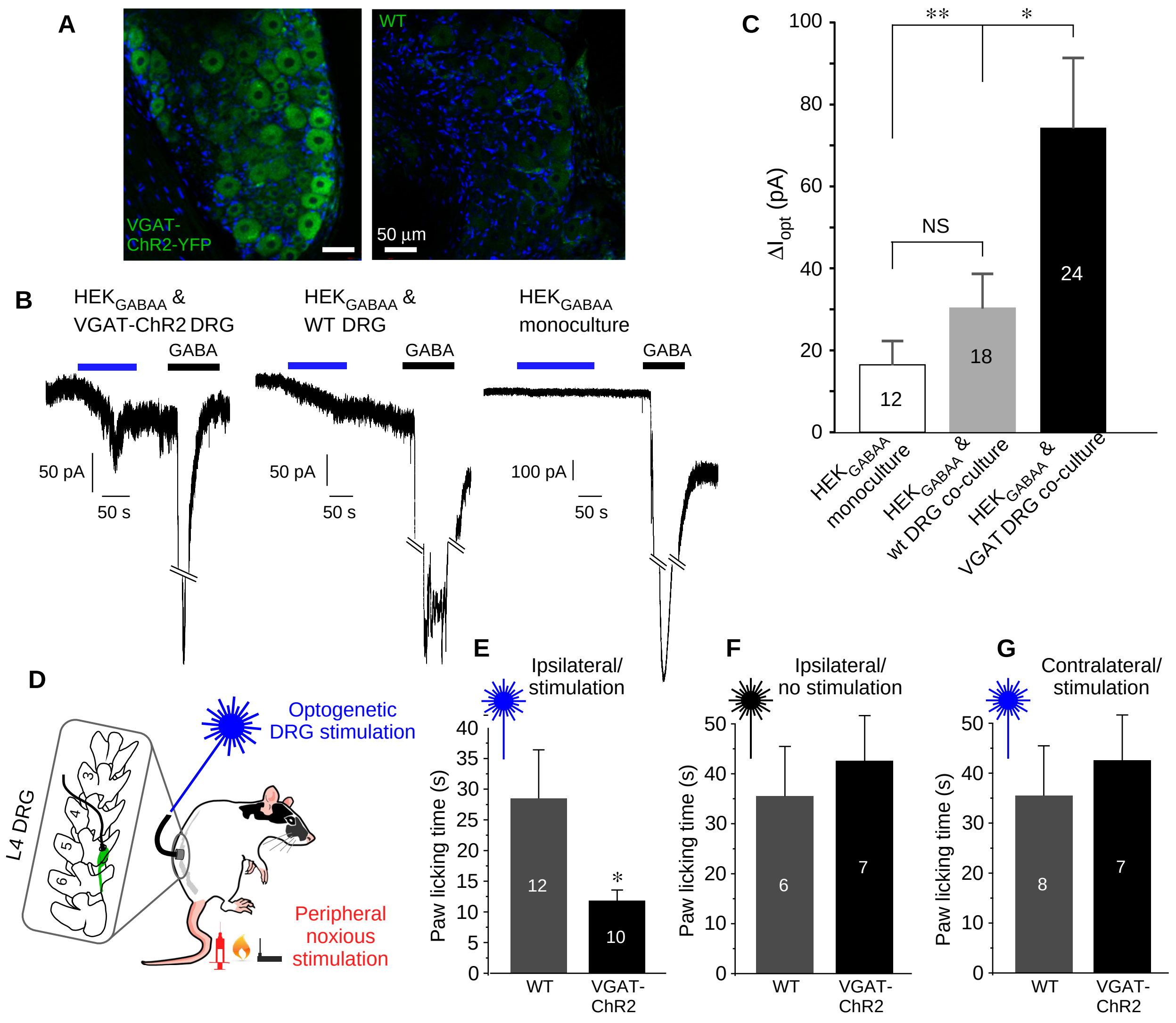

Supplementary Figyre 7. Optogenetic stimulation induces GABA release from DRG neurons and suppresses nociceptive transmission. A DRG neurons from the VGAT-ChR2-EYFP mice express FP. **B** Sniffing patch experiments similar to that shown in Fig. 1**A-E**; DRG neurons form VGAT-ChR2-EYFP mice (left) or from wild-type mice (middle) were co-cultured with HEK_{GABAA} cells; neurons were stimulated with 473 nm blue light (horizontal blue bars) and current responses were recorded from juxtaposed sniffing HEK_{GABAA} cells; 200µM GABA was applied at the end of experiment. Example on the right shows recording from HEK_{GABAA} cells in monoculture. Panel **C** summarizes the data shown in **B**. *,** indicate significant difference from the appropriate control at $p<0.05$ and $p<0.01$, respectively (Kruskal-Wallis ANOVA; Mann-Whitney test). **D**-G show i*n vivo* optogenetic experiments. **D** Schematic of the optical fiber implant and stimulation. **E-G** Effect of optical stimulation of DRG in VGAT-ChR2-EYFP mice on the nocifensive behaviour induced by hind-paw injection of BK (200µM; 50µl). **E** shows the effect of blue light stimulation on the paw licking produced by BK injection into the ipsilateral paw in WT and VGAT-ChR2-EYFP mice. * indicates significant difference from the WT control at $p<0.05$ (unpaired, two-tailed t-test). **F** shows experiment similar to that in **E** but without blue light stimulation. **G** is similar to **E** but BK was injected to the contralateral paw. In **C**, **E-G** the number of experiments is indicated within the bars.
